# APE1 binds and processes abasic sites present in i-motif DNA and cooperates with PCBP1 in maintenance of telomeric stability

**DOI:** 10.1093/nar/gkag686

**Published:** 2026-07-08

**Authors:** Alessia Bellina, Matilde Clarissa Malfatti, Tobias Obermann, Kayla Mae Grooms, Andreas Gjøsæther, Zahraa Othman, Gilmar Salgado, Daniela Marasco, Antonella Virgilio, Veronica Esposito, Giulia Antoniali, Catia Mio, Matteo Pivetta, Magnar Bjørås, Barbara van Loon, Gianluca Tell

**Affiliations:** Laboratory of Molecular Biology and DNA Repair, Department of Medicine, University of Udine, Piazzale Massimiliano Kolbe 4, 33100 Udine, Italy; Laboratory of Molecular Biology and DNA Repair, Department of Medicine, University of Udine, Piazzale Massimiliano Kolbe 4, 33100 Udine, Italy; Fondazione Italiana Fegato - ONLUS, Liver Cancer Unit, Trieste, Basovizza 34149, Italy; Department of Clinical and Molecular Medicine, Faculty of Medicine and Health Sciences, Norwegian University of Science and Technology, 7491 Trondheim, Norway; Department of Clinical and Molecular Medicine, Faculty of Medicine and Health Sciences, Norwegian University of Science and Technology, 7491 Trondheim, Norway; Department of Clinical and Molecular Medicine, Faculty of Medicine and Health Sciences, Norwegian University of Science and Technology, 7491 Trondheim, Norway; Department of Life Sciences and Technology for Health, ARNA Laboratory, INSERM U1212, CNRS, UMR 5320, University of Bordeaux, Bordeaux F-33076, France; Department of Life Sciences and Technology for Health, ARNA Laboratory, INSERM U1212, CNRS, UMR 5320, University of Bordeaux, Bordeaux F-33076, France; Department of Pharmacy, University of Naples Federico II, Via D. Montesano 49, 80131 Naples, Italy; Department of Pharmacy, University of Naples Federico II, Via D. Montesano 49, 80131 Naples, Italy; Department of Pharmacy, University of Naples Federico II, Via D. Montesano 49, 80131 Naples, Italy; Laboratory of Molecular Biology and DNA Repair, Department of Medicine, University of Udine, Piazzale Massimiliano Kolbe 4, 33100 Udine, Italy; Department of Medicine, University of Udine, Piazzale Massimiliano Kolbe 4, 33100 Udine, Italy; Department of Medicine, University of Udine, Piazzale Massimiliano Kolbe 4, 33100 Udine, Italy; Department of Clinical and Molecular Medicine, Faculty of Medicine and Health Sciences, Norwegian University of Science and Technology, 7491 Trondheim, Norway; Centre for Embryology and Healthy Development, University of Oslo, Oslo 0373, Norway; Department of Microbiology, Oslo University Hospital and University of Oslo, Oslo 0424, Norway; Department of Clinical and Molecular Medicine, Faculty of Medicine and Health Sciences, Norwegian University of Science and Technology, 7491 Trondheim, Norway; Laboratory of Molecular Biology and DNA Repair, Department of Medicine, University of Udine, Piazzale Massimiliano Kolbe 4, 33100 Udine, Italy

## Abstract

Apurinic/apyrimidinic endodeoxyribonuclease 1 (APE1) is a key enzyme in the base excision repair pathway, responsible for processing abasic (AP) sites. Recent studies revealed that APE1 participates in repairing DNA secondary structures as G-quadruplexes (G4). Telomeres, stabilized by shelterin proteins, are rich in G4, where APE1 binds and repairs AP-sites to maintain telomere integrity. The G4-complementary, cytosine-rich strand forms the i-motif (iM) structure, essential for telomere maintenance, though its repair mechanism remains unclear. Herein, we investigated APE1 binding and processing capabilities toward native and damaged telomeric iM, bearing AP-sites in different positions. Using biochemical and biophysical assays, we found that APE1 binds the telomeric iM sequence and that its cleavage efficiency depends on AP-site position within iM. Proximity ligation assay analysis, in HeLa and U2OS cells, highlighted a novel interaction between APE1 and PCBP1, a well-known iM-folding modulator. PCBP1 binds iM with higher affinity than APE1 and inhibits its cleavage activity on damaged iM. Immunofluorescence and telomere restriction fragment analyses showed that depletion of APE1 or PCBP1 impairs their interaction with the shelterin components, affecting telomere length. These results connect APE1 canonical DNA repair activity with the maintenance of non-canonical DNA secondary structures in telomeres, through its interaction with PCBP1.

## Graphical Abstract

**Graphical Abstract:**
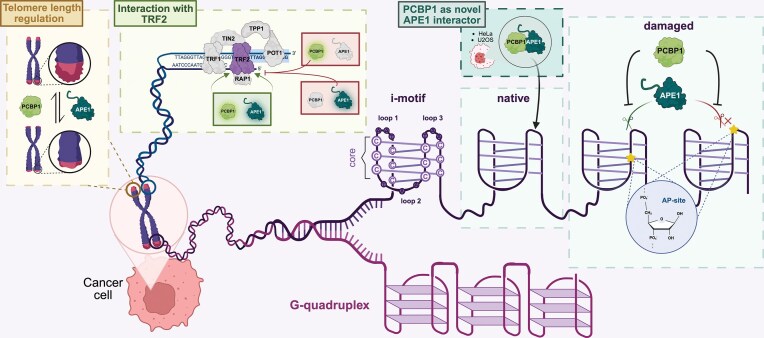

## Introduction

Intercalated motif (iM) DNA structures consist of two parallel-stranded duplexes intercalated in antiparallel orientation and held together by hemi-protonated cytosine-cytosine^+^(C:C^+^) base pairs [1]. The iM core is always composed of a scaffold of C:C^+^ base pairs, which varies in length between several iM, and by the loop regions connecting the core, which are specific for each sequence, differing in length and nucleotide composition [2]. It is well established that the formation of iM is dependent on acidic pH *in vitro* [1], while it might occurr also at physiological pH *in vivo* [3, 4], specifically under conditions of molecular crowding [5, 6], negative superhelicity induced by transcription [7], and in the presence of silver and copper cations [8, 9]. Through the Quadparser algorithm [3], sequences having four tracts of five cytosines, separated by 1–19 nt and able to form iM, were searched in the human genome [3], and surprisingly 5125 potential iM sequences were identified [3], with a 12.4% of them prevalently located in gene promoters. Through a bioinformatic analysis with the R package Biostrings, it has been demonstrated the presence of 769 dC_n_ sequences, with *n* between 15 and 81 nt, mainly at promoter levels, introns and 5′ and 3′ UTR [4]. Moreover, by using a specific synthetic antibody fragment (iMab), which recognizes iM with high selectivity and affinity in cells, it has been discovered that the number of iM foci dynamically changes during cell cycle progression [10]. Specifically, the highest number of foci formation occurs during the G1/S phase boundary with a consequent decrease in S phase, suggesting that iM are resolved before DNA replication with a role in regulating transcriptional activity [10]. The most relevant demonstration of an iM-regulatory role on DNA transcription concerns *BCL2* [11] and *HRAS* oncogenes [12]. Regarding *BCL2*, its promoter folds into a harpin or an iM, which are in a dynamic equilibrium. The ribonucleoprotein human ribonucleoprotein (hnRNP)-L binds to its iM, causing its unfolding to a single strand, thus leading to the activation of the *BCL2* gene transcription [11]. Similarly, the ribonucleoprotein hnRNP A1 is able to unfold the iM present in the promoter of *HRAS*, leading to the activation of the *HRAS* gene transcription [12]. Many studies demonstrated the presence of iMs also within telomeres [10, 13, 14], which are the protective ends of the chromosomes formed by 5–15 kb long tracts of double-stranded (ds) guanine and cytosine repeats, with a 50–300-nt protrusion of single-stranded (ss) guanine repeats at the 3′end, called G-tail or G-overhang [15, 16]. Under physiological conditions, telomeres shorten every cell division until reaching a critical length that triggers intracellular senescence signals [16, 17]. This highly repetitive and GC-rich nature of telomeres is strictly tied with their capability of forming high-order DNA secondary structures, such as G-quadruplex (G4) and iM [18]. In detail, in U2OS cell line, a co-localization signal between the shelterin-complex protein Telomeric repeat-binding factor 2 (TRF2) and iM foci was observed [10], confirming the presence of iM structures at the telomeric level, with potential relevant biological roles. After a treatment with an iM stabilizing ligand (i.e. single-walled carbon nanotubes—SWNT), the activity of telomerase was hindered, preventing the extension of telomeric DNA and leading to defective telomere maintenance [13]. In addition, this treatment caused telomere uncapping, characterized by high levels of anaphase bridges and micronuclei, the release of telomere-binding proteins such as TRF2 and protection of telomeres protein 1 (POT1), which induces telomere malfunction and DNA damage signaling, with a consequent phenotype characterized by cell cycle arrest, apoptosis, and senescence in HeLa cells [14]. *In vitro* experiments demonstrated that both unstructured telomeric C-strands and telomeric iM inhibit the processivity of telomerase extension of parallel G4 and linear telomeric DNA [19]. Recently, it has been proposed that iM structural dynamics can be modulated by a specific protein family, called poly(C)-binding proteins (PCBPs) [11, 12, 20–22]. In particular, one member of this family, PCBP1 (also called hnRNP E1) was reported to be involved in the competitive formation of iM and G4 structures in NMuMG and A549 cell lines and in the maintenance of genomic integrity. Specifically, PCBP1 co-localized with iM, and its knockdown led to a decreased accumulation of iM foci in the nucleus and the induction of DNA damage signaling, which is very high upon genotoxic treatments [23]. Furthermore, cell treatment with SWNT influenced PCBP1 localization, delocalizing it from telomeres as TRF2 and POT1 [14].

At present, there is no evidence whether lesions occurring on iM may affect their structural and functional properties and, above all, which proteins are responsible for the repair of damaged iM DNA structures. Apurinic/apyrimidinic (AP) sites are among the most frequent lesions in cells with almost 10 000 sites per human cell per day [24]. They can be generated spontaneously or enzymatically by the action of DNA glycosylases on damaged bases, such as oxidized bases [24]. AP sites are recognized and processed by the apurinic/apyrimidinic endodeoxyribonuclease 1 (APE1), the principal enzyme of the base excision repair (BER) pathway [25]. Several studies, performed by our laboratory and others, showed that APE1 holds a highly substrate- and structure-dependent endonuclease activity [26, 27], particularly in the context of non-canonical secondary structures. Indeed, APE1 activity is lower on damaged telomeric G4 structures compared to the canonical ds substrates, and strongly depends on: (i) the position of the damaged base, (ii) the N-terminal region of the protein, and (iii) the ionic strength of the reaction [28–30]. Differently from G4 structures, which have been widely studied in the last years [28–31], the role of APE1 on their iM counterparts has not been investigated, yet. Recently, Dvorakova and colleagues studied how natural base lesions (i.e. AP sites, uracil, 8-oxo-adenine) impact telomeric iM formation, revealing that modifications in the loops may cause minor alterations in the iM formation and stability, while modifications in the core have a more extensive effect, with different consequences depending on their position and abundance [32]. On this basis, this study was aimed at characterizing the effects of AP sites on the folding of telomeric iM structures, the processing activity of APE1 on damaged structures, and the effect of PCBP1 protein on APE1 activities. Using complementary biochemical, biophysical, and cellular investigations, we showed that: (i) APE1 is able to stably bind the telomeric iM structure; (ii) AP-containing telomeric sequences can fold into iM structures, and AP sites affect the iM folding and stability according to their position in the structure; (iii) APE1 cleavage activity on AP sites strongly depends on the AP position and on APE1 N-terminal region; (iv) APE1 and PCBP1 are found in close proximity in HeLa and U2OS cellular lines; (v) PCBP1 inhibits APE1 cleavage on telomeric iM sequence; (vi) the depletion of APE1 and PCBP1 dysregulates telomere length with opposite trends in a Alternative Lengthening of Telomeres (ALT)-U2OS model; (vii) APE1 and PCBP1 can mutually and dependently interact with the shelterin protein TRF2; (viii) APE1 depletion correlates with a diminishment of iMab foci, while PCBP1 depletion with an increase of iMab foci in U2OS cells; (ix) APE1 and PCBP1 depletion leads to DNA damage response (DDR) activation in U2OS cellular model, with a possible radical alteration of DNA damage signaling when they are both depleted. Our study, besides deeply characterizing a still unexplored field in DNA repair mechanisms of BER enzymes, opens novel perspectives for translational applications in biology and medicine, thus further extending BER function in telomere biology.

## Materials and methods

### Protein expression and FPLC purification

*Escherichia coli* BL21 (DE3) bacteria (C2530H, New England Biolabs) were transformed with 100 ng of plasmids pGEX-3X APE1^WT^, pGEX-3X APE1^N∆33^, pGEX-3X APE1^H309N^, PCBP1_Full-length_pGEX, (Addgene, 135108, [33]), pTAC-MAT-APE1^WT^-GFP, pTAC-MAT-APE1^N∆33^ -GFP, pTAC-MAT-APE1^E96A^, or pET166-E1 for the expression of GST-tagged APE1^WT^, APE1^N∆33^, APE1^H309N^, PCBP1, His- and GFP-tagged APE1^WT^, APE1^N∆33^, and His-tagged APE1^E96A^ and PCBP1, respectively. The bacterial culture was harvested at 37°C at 250 rpm. After reaching an OD_600 _= 0.6, the protein expression was induced with 1 mM IPTG (11411446001, Roche), and bacteria were left to grow for 4 h at 37°C. Pellets were then lysed in the presence of a protease inhibitors cocktail (P8465, Merck) and lysozyme through six cycles of sonication of 30 s. The sample was then centrifuged at 23 000 × *g* for 20 min at 4°C, and the supernatant was conserved and filtered with a 0.45 µm filter for the following FPLC purification (GE Healthcare). Purification of the GST-tagged recombinant proteins was carried out using GSTrap columns (GE17-5281-01, GE Healthcare) following the manufacturer’s instructions. Protein elution was obtained by 10 mM GSH (G4251, Merck). The GST tag was also removed through incubation with Factor Xa (F9302, Merck) following the manufacturer’s instructions, and the recombinant protein was separated from the tag by using a HiTrap Benzamidine HP column (GE17-51243-01, GE Healthcare). The purification of the His-tagged recombinant proteins was carried out using an HisTrap HP [IMAC(Ni^2+^)] column (GE17-5247-01, GE Healthcare), and they were eluted by setting an imidazole gradient, with a final concentration of 800 mM. All protein fractions were dialyzed and stored at −80°C in 25 mM Tris–HCl (pH 7.5), 100 mM NaCl, 10% glycerol.

### Oligonucleotides and annealing

All the oligonucleotides (ODNs) used in this study are reported in Table 1. The ODNs were synthesized by Metabion and Ella Biotech, purified by HPLC, and checked in mass spectrometry. Where indicated, the ODN holds a tetrahydrofuran base that substitutes the canonical nucleotide for mimicking an abasic site (AP), an IRDye-800, or a quencher BBQ650 at the 5′. The ODNs were resuspended in DNase- and RNase-free water, at a final concentration of 100 µM. To allow iM formation, 100 pmol of each ODN were prepared in a final volume of 40 μl with 50 mM Tris-acetate (pH 5.5) and 50 mM KCl, and allowed to sit o/n at RT. For the experiments with ds ODNs, 100 pmol of each ODN were annealed with 150 pmol of its complementary DNA oligonucleotide in 10 mM Tris–HCl (pH 7.4) and 10 mM MgCl_2_, heated at 95°C, and cooled down o/n in the dark. For the Lumicks C-trap approach, DNA iM substrate, called 3×-C-NAT, was purchased from Ella Biotech, and its sequence is reported in Table 1. The substrate was ligated inside long handles by using the Hairpin Labeling and Tethering Kit (Lumicks, 00016/17). Long handles are derivatized with three biotins and three digoxigenins at the opposite ends to allow the tethering by using streptavidin and anti-digoxigenins beads.

**Table 1.** Sequences of the ODNs used in this study

Name	Sequence (5′–3′)	Length (nt)	Length of the product (nt)	Modification at 5′
ss_dF	GGATCCGGTAGT**(AP)**TTAGGCCTGAAC	25	12	IRD800
ss_dC	GTTCAGGCCTAACACTACCGGATCC	25	-	-
C-NAT	TAACCCTAACCCTAACCCTAACCCTAA	27	-	IRD800
AP14	TAACCCTAACCCT**(AP)**ACCCTAACCCTAA	27	13	IRD800
AP20	TAACCCTAACCCTAACCCT**(AP)**ACCCTAA	27	15	IRD800
AP16	TAACCCTAACCCTAA**(AP)**CCTAACCC TAA	27	16	IRD800
AP17	TAACCCTAACCCTAAC**(AP)**CTAACCCTAA	27	19	IRD800
ss_dG	TTAGGGTTAGGGTTAGGGTTAGGGTTA	27	-	-
C-NAT-helix	TAACCCTAACCCTAACCCTAACCCTAATTTATCA GTACTTGTCAACACGA			
	GCAGCCCGTATATTCTCCTACAGCACTA	78	-	-
AP14-helix	TAACCCTAACCCT**(AP)**ACCCTAACCCTAATTTAT CAGTACTTGTCAACACGA			
	GCAGCCCGTATATTCTCCTACAGCACTA	78	13	-
AP16-helix	TAACCCTAACCCTAA**(AP)**CCTAACCCTAATTTAT CAGTACTTGTCAACACGA			
	GCAGCCCGTATATTCTCCTACAGCACTA	78	15	-
AP17-helix	TAACCCTAACCCTAAC**(AP)**CTAACCCTAATTTATC AGTACTTGTCAACACGA			
	GCAGCCCGTATATTCTCCTACAGCACTA	78	16	-
AP20-helix	TAACCCTAACCCTAACCCT**(AP)**ACCCTAATTTATC AGTACTTGTCAACACGA			
	GCAGCCCGTATATTCTCCTACAGCACTA	78	19	-
C-NAT-helix-BBQ	TAACCCTAACCCTAACCCTAACCCTAATTTATCA GTACTTGTCAACACGA			
	GCAGCCCGTATATTCTCCTACAGCACTA	78	-	BBQ650
AP14-helix-BBQ	TAACCCTAACCCT**(AP)**ACCCTAACCCTAATTTA TCAGTACTTGTCAACACGA			
	GCAGCCCGTATATTCTCCTACAGCACTA	78	13	BBQ650
AP16-helix-BBQ	TAACCCTAACCCTAA**(AP)**CCTAACCCTAATTTAT CAGTACTTGTCAACACGA			
	GCAGCCCGTATATTCTCCTACAGCACTA	78	15	BBQ650
AP17-helix-BBQ	TAACCCTAACCCTAAC**(AP)**CTAACCCTAATTTAT CAGTACTTGTCAACACGA			
	GCAGCCCGTATATTCTCCTACAGCACTA	78	16	BBQ650
AP17-helix-BBQ	TAACCCTAACCCTAACCCT**(AP)**ACCCTAATTTATC AGTACTTGTCAACACGA			
	GCAGCCCGTATATTCTCCTACAGCACTA	78	19	BBQ650
3× - C-NAT	TAACCCTAACCCTAACCCTAACCCTA ACCCTAACCCTAACCC			
	TAACCCTAACCCTAACCCTAACCC TAACCCTAA	75	-	-

The names of the ODNs is reported in the first column. The sequence of the 5′–3′ ss ODNs are reported in the second column. The modified bases are highlighted in bold type. The whole length and the length of the product are reported in the third and fourth columns, respectively. In the fifth column, the modification at the 5′ of each ODN is reported.

### Circular dichroism spectroscopy

Circular dichroism (CD) samples of ODNs reported in Table 1 were prepared at an ODN concentration of 6 µM by using a buffer with 50 mM Tris-acetate, 50 mM KCl, and pH 5.5, and submitted to the annealing procedure by heating at 70°C and slowly cooling to room temperature. CD spectra of all iM structures and CD melting curves were registered on a Jasco 715 CD spectrophotometer (Jasco, Tokyo, Japan). For the CD spectra, the wavelength was varied from 360 to 220 nm at 100 nm min^−1^ scan rate, and the spectra were recorded with a response of 4 s at a 2.0 nm bandwidth, and normalized by subtraction of the background scan with buffer. The temperature was kept constant at 10°C with a thermoelectrically controlled cell holder (PTC-348, Jasco). CD melting curves were registered as a function of temperature from 10°C to 80°C for iM-forming ODNs at their maximum Cotton effect wavelengths. The CD data were recorded in a 0.1 cm pathlength cuvette with a scan rate of 0.5°C/min.

### NMR spectroscopy

NMR samples were prepared in a buffer containing 50 mM NaCl, 10 mM MES (pH 5.5), and 1 mM 1,4-Dithiothreitol (DTT), unless otherwise indicated. All measurements were acquired at 25°C in 3 mm thin-wall NMR tubes with 5% D_2_O. Recombinant APE1 was uniformly 15^N^-labeled and dissolved in the same buffer at a concentration of 100 µM. The C-NAT oligonucleotide was dissolved in an identical buffer and annealed by heating to 95°C for 5 min, followed by slow cooling to RT. For titration experiments, the C-NAT was added stepwise to the 15^N^-labeled UP1 solution at defined molar ratios.

NMR spectra were recorded on a Bruker 800 MHz AVANCE NEO spectrometer (IECB, Bordeaux, France) equipped with a TCI cryoprobe. One-dimensional 1H spectra were acquired using the zgesgppe pulse sequence, and two-dimensional 1H–15N correlation spectra were obtained with the trosyett3gpsi sequence. Spectra were collected for APE1 alone and after successive additions of the C-NAT. Data were processed with TopSpin 4.1 (Bruker Biospin) using standard procedures. Chemical shift perturbations were calculated as weighted combined differences in 1H and 15N shifts according to the equation:


(1) \begin{eqnarray*}
{\mathrm{\delta = }}\sqrt {{\mathrm{\delta }}{{\mathrm{H}}}^2 + \left( {{{\left( {{\mathrm{\delta N/5}}} \right)}}^2} \right)} .
\end{eqnarray*}

Assignments were transferred from published APE1 datasets (PDB code: 1bix), and perturbation analyses were used to identify residues involved in iM binding, with δH the shift in 1H dimension and δN the shift in 15N dimension. The most significant shifts were depicted on the surface of APE1 using UCSF ChimeraX.

### SwitchSENSE technology

SwitchSENSE experiments were performed on a HeliX^®^ instrument (Dynamic Biosensors GmbH) on standard switchSENSE adapter chips (ADP-48-2-0, Dynamic Biosensors GmbH) using the static measurement mode. The experimental workflow was designed in the HeliOS software (Version 2024.2.1, Dynamic Biosensors GmbH). To investigate the interaction between iM and APE1, the adapter chips were first functionalized. On the chip surface, a DNA construct called nanolever is attached. The part of the nanolever that is connected with the chip is the anchor strand and is extended by the adapter strand. The 3′ end of the adapter strand is derivatized with a fluorescent dye (Ra). This dye is used as the read out and is excited in the range of 600–630 nm, and emission is recorded in the range of 650–680 nm. The complementary sequence of the adapter strand is annealed with its counterpart, which toward the 5′ end is extended by our sequences of choice. Here, two new sets of ODNs (Table 1) were synthesized (Ella Biotech). One set of ODNs was modified with a quencher (BBQ650) at the 5′ end of the oligonucleotide and was exclusively used for iM-folding experiments. To account for the nonspecific binding of APE1 toward dsDNA, the adapter chips have a reference spot. Here, the nanolever setup is similar, except that no overhang was designed. Hence, the nanolever is in dsDNA configuration, and the signal recorded serves as a reference. Also, for the iM-folding experiments where the quencher was employed, the nanolever was in a dsDNA configuration to account for pH-dependent quenching.

iM-forming ODNs were hybridized with adapter strand 1-Ra at 1:1 ratio (v/v) trough incubation at 25°C for 30 min at 650 rpm. For the reference, adapter strand 2, prehybridized with the complementary sequence, was then added at a 1:1 ratio (v/v). Experiments were carried out in MES200 buffer [10 mM MES; 200 mM NaCl; 50 µM ethylenediaminetetraacetic acid (EDTA); 50 µM Ethyleneglycol- bis(β-aminoethyl)-N,N,Nʹ,Nʹ-tetraacetic Acid (EGTA); 0.05% Tween-20], at a pH of 5.5 or else specified. Two different principal approaches were used for the SwitchSENSE experiments. The first one relies on the iM recognition by a ligand, which leads to changes in the dye environment and in the fluorescence signal. For the iM folding validation, the used ligand was the iMab antibody [10]. For the binding assays, APE1^WT^-GFP and APE1^N∆33^-GFP were used as ligands. Here, the different ligands have been applied in different concentrations to obtain a dose-dependent response curve. Additionally, blanks containing only the MES200 buffer were ran before and after the ligand runs. After every concentration, the chip surface was regenerated using 6 M guanidinium hydrochloride and then was functionalized with a new nanolever. Thereby, we ensured that all ligand is stripped off from the chip surface. To be able to obtain the desired kinetic values, every measurement consists of an association phase and a dissociation phase. During the association phase, the respective ligand is injected onto the chip at a flow rate of 200 µl/min. In the dissociation phase, the chip surface is washed with the MES200 buffer at a flow rate of 500 µl/min. The chip temperature is set to 25°C, while the sample tray was cooled to 10°C.

The second approach relies on the presence of BBQ650 at the 5′ end of the oligonucleotide. Upon iM folding, the quencher is physically close to the dye of the complementary strand and induces a quenching signal. By subsequently injecting buffers with increasing pH (association phase), it can be determined if the quencher is still in close proximity to the dye. After having determined the iM formation by dye quenching, unfolding is induced by injecting a high-pH solution; consequently, the quencher is led far away, which is represented by fluorescence increase. Parameters set during the association and dissociation phases are as described before. Chip temperature was as well set to 25°C, and sample tray was at RT.

To obtain kinetic curves representing ligand–iM interaction, removing nonspecific binding to the double-stranded nanolever on spot 2, the obtained fluorescence signals for the iM were referenced against spot 2. Additionally, the blanks were used to set a stable baseline. The chosen fit model was individually chosen based on the generated data.

In case of the quencher experiments, spot 2 served for a reference for pH-dependent quenching. iM formation was measured in percent fluorescence change.

### SDS–PAGE, western blot, and southwestern blot analysis

Recombinant proteins and cell extracts were loaded on an sodium dodecyl sulfate–polyacrylamide gel electrophoresis (SDS–PAGE) gel for electrophoresis. For the quantification of recombinant proteins, each band was quantified and normalized with a known-concentration BSA (bovine serum albumin) standard curve. The staining of the gel was performed with Coomassie Blue.

For the western blot analysis, upon running the gel, the proteins were transferred to a nitrocellulose membrane (Amersham^TM^ Protran^®^ 0.2 µm NC, GE10600001, Merck). Protein transfer was performed in Towbin buffer [trizma 25 mM, glycine 23 mM, methanol 20% (v/v)] at constant 70 V for 3 h at 4°C. The membrane was colored with Revert 700 Staining (926-11010, Li-Cor Biosciences) and acquired with Odyssey CLx scanner/ImageStudio Software (Li-Cor Biosciences). The membrane was blocked for 1 h with 5% BSA in phosphate buffered saline (PBS)-Tween 0,1% and then incubated with primary antibody for 3 h at RT. The primary antibodies used and their dilution usage were the following: APE1, mouse, monoclonal, 1:2000, NB 100-116, Novus; APE1, rabbit, polyclonal, 1:2000, NB 100-101, Novus; PCBP1, mouse, 1:500, sc-137249, Santa Cruz Biotechnology; PCBP1, rabbit, polyclonal, 1:2000, NBP2-55063, Novus; Tubulin, mouse, monoclonal, 1:2000, T0198, Merck; p21, rabbit, polyclonal, 1:500, 2947, Cell Signaling; *γ*H2AX, mouse, monoclonal, 1:500, 05-636, Merck; TRF2, mouse, monoclonal, sc-271710, Santa Cruz Biotechnology; HSP70, rabbit, polyclonal, 1:2000, GTX111088, GeneTex. Then, the membrane was incubated with secondary antibodies labeled with IRDye 700 or 800 (1:10 000, 926-68 071, Li-Cor Biosciences).

For the southwestern blot (SWB) analysis, 2 µg of each protein was loaded on the SDS–PAGE gel. After running and blotting, the membrane was incubated for 10 min at room temperature in a 6 M guanidine hydrochloride solution to denature the proteins. The proteins were then renatured by incubating the membrane with serial guanidine hydrochloride dilutions until 0.094 M in SWB Buffer (10 mM HEPES, 50 mM NaCl, 10 mM MgCl_2_, 0.1 mM EDTA, 1 mM DTT, 50 μM ZnSO_4_, and 0.1% Tween-20) for 10 min at 4°C. The membrane was then washed twice with SWB buffer and then incubated for 1 h at RT with SWB blocking buffer (5% BSA in SWB buffer). At the end, the membrane was incubated with 5 pmol of the fluorescent oligonucleotide in SWB blocking buffer at 4°C with gentle rocking o/n, washed with SWB buffer and analyzed.

All images were acquired by an Odissey CLx scanner (Li-Cor Biosciences) and analyzed by ImageStudio Software (Li-Cor Biosciences).

### Binding assay by UV-crosslinking

For the UV-crosslinking analysis, the reactions were prepared in a final volume of 20 μl with the indicated doses of APE1^WT^, APE1^N∆33^, or PCBP1 recombinant proteins and 25 nM of each ODN, in a buffer containing 50 mM Tris-acetate pH 5.5, 50 mM KCl, 1 mM DTT, 0.8% glycerol, 400 µM EDTA. The reactions were incubated for 1 h and 30 min at 4°C and then UV-crosslinked at 0.2 J/m^2^. The samples were then added with 6.6 µl of Laemmli (2 M Tris–HCl, pH 6.8, 20% SDS, 14 M β-mercaptoethanol, 10% glycerol, and traces of bromophenol blue) and heated at 95°C for 5 min. The samples were run on a 12% SDS–PAGE gel. After running, the gels were acquired by Odyssey CLx scanner and analyzed by ImageStudio Software (Li-Cor Biosciences).

### AP-site incision assay

To measure the endonuclease activity of recombinant proteins, we performed AP-site incision assay as described in [34, 35]. Briefly, the oligonucleotide substrates (25 nM) were incubated at 37°C with different amounts of proteins and for different timing points, as indicated in the legend of each figure. Reactions were carried out in a buffer containing 20 mM Tris–HCl, pH 7.4, 50 mM KCl, 1 mM MgCl_2_, 0.1% BSA (v/v), and 0.1% Tween-20 (v/v). At the end, reactions were blocked with a stop solution [99.5% (v/v) formamide, 10× Orange Loading Dye (927-10100, Li-Cor Biosciences)] and heated at 95°C for 5 min. Samples were loaded onto a 7 M urea denaturing 20% polyacrylamide gel in TBE buffer (45 mM Tris, 45 mM boric acid, 1 mM EDTA, pH 8.0) and run at 4°C at 300 V for 30 min. The gel was directly scanned by Odyssey CLx scanner (Li-Cor Biosciences) and analyzed by ImageStudio Software (Li-Cor Biosciences).

### Cell culture, CRISPR/Cas9, transfection, and nuclear cell extract preparation

For the experimental procedures performed in mammalian cells, different human tumoral adherent cell lines were used, including HeLa cells and APE1 inducible knock-down HeLa clones (SCR and Cl.3) from cervical tumor, U2OS cells from bone osteosarcoma, and an APE1^WT^-mEGFP-expressing A549 stable clone (clone 31) from lung adenocarcinoma. HeLa and U2OS were grown in Dulbecco’s modified Eagle’s medium (DMEM) High Glucose medium (ECB7501L, EuroClone) complemented with 10% (v/v) fetal bovine serum (FBS), penicillin (100 U/ml), streptomycin (100 mg/ml), and L-glutamine (2 mM) (EuroClone). HeLa SCR and Cl.3 were grown in DMEM High Glucose medium (ECB7501L, EuroClone) complemented with 10% (v/v) of FBS, penicillin (100 U/ml), streptomycin (100 mg/ml), L-glutamine (2 mM) (EuroClone), 3 µg/ml blasticidin, 100 µg/ml zeocin, and 400 µg/ml geneticin (Invitrogen). For inducible knock-down of APE1, doxycycline (1 µg/ml, Merck) was added into the medium, and cells were cultured for 10 days [36, 37]. A549 clone 31 cells were grown in RPMI 1640 medium (ECM9106L, EuroClone) complemented with 25 mM HEPES, 10% (v/v) FBS, penicillin (100 U/ml), streptomycin (100 mg/ml), and L-glutamine (2 mM) (EuroClone). All cells were grown in an incubator at a constant and monitored temperature of 37°C and a partial pressure of CO_2_ of 5%.

The A549 clone 31 cell line was generated using the strategy previously described in [38] and deeply characterized in the Supplementary section of this article. Briefly, APE1^WT^-mEGFP-expressing cells were generated by transiently transfecting A549 cell line with Lipofectamine 3000 (L3000001, Life Technologies) through puC19 repair template for the tagging of APE1 C-term at endogenous loci, together with two plasmids encoding for two single guide RNAs, following the manufacturer’s instructions. Cells were expanded for 7 days before FACS sorting with Multi-Application Cell Sorter MA900 (Sony Biotechnology Inc.) based on the mEGFP signal. All cells mEGFP^+^ were then clonally expanded, and characterization of single-cell clones was performed by immunoblotting and Sanger sequencing.

For U2OS transfection, 2 million cells were plated in a 10-mm Petri. The day after, cells were transfected either with 15 µg of pCMV6-Entry (PS100001, Origene) as a control, or pCMV6-ENTRY PCBP1 (RC207878, Origene), with 50 µl of Lipofectamine 2000 (116668-019, Invitrogen) in 4.15 ml of OPTIMEM (3185070, Thermo Fisher) for 4 h at 37°C. Next, cells were washed with PBS 1×, and fresh medium was added. After 48 h of transfection, cells were used for experimental procedures. For the HeLa and U2OS silencing, 300 000 cells were plated in a six-multiwell. The day after, cells were transfected with 100 pmol of scramble (5′ - CCA UGA GGU CAG CAU GGU CUG UU - 3′, siSCR) as a negative control, 100 pmol of ON-TARGETplus Human PCBP1 SMART pool (siPCBP1), and 100 pmol of siAPE1 (5′ - UACUCCAGUCGUACCAGACCU - 3′) (T-2005-01, Dharmacon), with 2.5 μl of Dharmafect (Dharmacon) in 200 μl of OPTIMEM (3185070, Thermo Fisher) for 6 h at 37°C. Next, 1 ml of fresh DMEM was added to the cells, without removing the complexes. After 24 h, cells were washed once with PBS 1× and left to grow in fresh medium.

Nuclear cellular extracts (NCEs) with endogenously tagged APE1-mEGFP were obtained from A549 clone 31 cell line derived from 4 million cells treated with the Nuclear Extraction Kit (ab113474, Abcam) and three cycles of 10-s sonication with Bioruptor Pico at 4°C. The resulting nuclear extracts were aliquoted into single-use tubes and flash-frozen in liquid nitrogen for then long-term storage at −80°C. NCE protein concentration was determined by using Bradford assay (5000201, Bio-Rad), while NCE nucleic acid concentration was determined using Quant-iT™ PicoGreen™ dsDNA Assay Kit (P7589, Invitrogen). The DNA concentration in the NCE was in range of 40 ng/µl.

### Single-molecule analysis of DNA-binding proteins from nuclear extracts

Single-molecule analysis of DNA-binding proteins from nuclear extracts (SMADNE) analysis was carried out on Dymo C-trap^®^ instrument (LUMICKS), by applying general principles described in [39]. C-trap^®^ technology unites a microfluidic system, a dual-tap optical system, and a three-color confocal fluorescence microscope. All five channels of the microfluidic flow cell were pressurized to 0.10 bar. Prior to experiments, the channel used for NCE was passivated with casein. Reagents were diluted in recording buffer (25 mM MES, pH 5.5, 150 mM NaCl, 0.1 mg/ml BSA, 1 mM DTT, 1 mM Trolox). In ascending order, channels were loaded with 1.18 μm streptavidin-coated beads, 0.81 μm anti-digoxigenin beads, ligated DNA iM substrate, recording buffer, and NCE (1:45). A single DNA tether was formed between optically trapped beads by adjusting the distance between them. The tether was washed in recording buffer, flow was stopped, and force–distance curves were compared to an extensible worm-like chain model to confirm single DNA tether formation and iM structure unfolding (∼22 pN). During recordings, DNA tension was maintained between 10 and 15 pN, and continuous confocal line scanning was initiated along the tether as it moved into the NCE channel. The EGFP fluorescence was excited by a 488-nm laser at 5% power, detected through a 525/45-nm emission filter, and captured using a 1.2 NA, 60× water immersion objective and single-photon avalanche photodiodes. Kymographs were generated with 20-nm pixel size, 0.2-ms pixel dwell time, and 0.062-s line time.

Kymographs were analyzed using a custom Python script based on Pylake (v1.5.3, LUMICKS). Binding events were tracked using a greedy algorithm [40] by manually selecting regions encompassing individual events lasting at least 240 ms. Tracks separated by <1 s at the same position were manually connected to account for eGFP blinking. Events within 0.1 μm of the DNA midpoint (site of iM) were classified as specific. Specific gap times were defined as intervals between consecutive specific binding events. Binding and gap times were analyzed by cumulative residence time distribution (CRTD) and cumulative gap time distribution (CGTD), respectively, using an exponential one-phase decay fit to obtain rate constants.

### Telomeric chromatin immunoprecipitation and quantitative PCR

Telomeric chromatin immunoprecipitation (telo-ChIP) was performed on U2OS cells, following 48 h of transfection with either pCMV6-Entry (PS100001, Origene), as control, or pCMV6-ENTRY PCBP1 (RC207878, Origene). Before harvesting, cells were washed with PBS 1× and collected by trypsination. After washing cells with PBS 1×, formaldehyde was added to a final concentration of 1% v/v, and cross-linking was performed for 5 min at RT on a rocker. Afterwards, glycine was added to a 125-mM final concentration, and cells were incubated for 2 min at RT on a rocker. After centrifugation, cells were washed twice with ice-cold PBS 1×. Then, cells were lysed in 400 µl of Lysis Buffer (1% v/v SDS, 10 mM EDTA, pH 8, 50 mM Tris–HCl, pH 8), complemented with 1× protease inhibitor cocktail, 0.5 mM phenylmethylsulfonyl fluoride, 1 mM NaF, and 1 mM Na_3_VO_4_, for 10 min on ice. The obtained lysates were sonicated with Bioruptor Plus (Diagenode) at maximum power for 20 times for 30 s, with breaks of 30 s at 4°C. The sonicated material was then centrifuged at 12 000 rpm for 15 min at 4°C, and the supernatant was diluted 10 times in ChIP dilution buffer (1% v/v Triton X-100, 2 mM EDTA, 20 mM Tris–HCl, pH 8, 150 mM NaCl), complemented with the same protease inhibitors listed earlier. Immunoprecipitation was carried out using 50 µl of Anti-FLAG M2 Affinity Gel (A2220, Merck) for each reaction. Diluted surnatants were complemented with 40 µg of BSA and 16 µg of salmon sperm DNA and then incubated with the resin o/n at 4°C on a rocker. The day after, samples were centrifuged at 8000 × *g* for 1 min at 4°C, and the flow-trough was discarded. The resin was then washed once with 1 ml of Low Salt Wash Buffer (0.1% v/v SDS, 1% v/v Triton X-100, 150 mM NaCl, 2 mM EDTA, 20 mM Tris–HCl, pH 8), High Salt Wash Buffer (0.1% v/v SDS, 1% v/v Triton X-100, 500 mM NaCl, 2 mM EDTA, 20 mM Tris–HCl, pH 8), LiCl Wash Buffer (1% NP-40, 1% Na-deoxycolate, 1 mM EDTA, pH 8, 10 mM Tris–HCl, pH 8, 0.25 M LiCl), and twice with TE Buffer (10 mM Tris–HCl, pH 8, 1 mM EDTA, pH 8). Each washing step was carried out on a rocker for 5 min at RT, followed by a centrifugation at 8000 × *g* for 1 min at 4°C. Elution was carried out twice in rotation by incubating the resin with Elution Buffer (1% v/v SDS, 100 mM NaHCO_3_) for 15 min. Input and IP samples were reverse-crosslinked by incubation with 20 µg of Proteinase K for 2 h at 68°C with 1300 rpm shaking. Recovered DNA was purified with phenol-chloroform extraction and precipitated with ethanol. Telomeric DNA sequences were amplified by quantitative polymerase chain reaction (qPCR) using SensiFAST SYBR No-ROX Kit (Bioline), as described in [28].

### Immunofluorescence, proximity ligation assay, and telomeric fluorescence *in situ* hybridization

For immunofluorescence analysis, 80 000 cells were plated on glass slides placed inside a 24-multiwell. After indicated treatment, cells were fixed with 4% paraformaldehyde (PFA) for 20 min at RT. For the detection of APE1 and PCBP1, cells were permeabilized using Triton X-100 0.25% in PBS 1× for 5 min at RT and blocked with FBS 10% in Wash Buffer A (WBA, 0.15 M NaCl, 0.01 M Tris, 0.05% m/v Tween 20, pH 7.5) for 30 min at RT. The slides were then incubated with the primary antibody (PCBP1, 1:50, sc-137249, Santa Cruz Biotechnology; APE1, 1:100, NB 100-101, Novus) diluted in FBS 10% in WBA at 37°C for 2 h. Next, cells were incubated with the secondary antibody (Alexa Fluor 488, 111-545-003, Jackson ImmunoResearch; Alexa Fluor 633, A211050, A-21071, Invitrogen; Alexa Fluor 555, ab150114, Abcam), diluted in FBS 10% in WBA for 1 h at RT. For the detection of TRF2, cells were permeabilized with permeabilization solution (20 mM Tris–HCl, pH 8.0, 50 mM NaCl, 3 mM MgCl_2_, 300 mM sucrose, 0.5% Triton X-100) for 15 min at RT and blocked with BSA 1% in PBS for 1 h at RT. The slides were then incubated with primary antibody (TRF2, 1:100, sc-271710, Santa Cruz Biotechnology) for 2 h at RT, diluted in BSA 1% in PBS. Finally, cells were incubated with the secondary antibody (Alexa Fluor 488, 111-545-003, Jackson ImmunoResearch; Alexa Fluor 633, A211050, A-21071, Invitrogen; Alexa Fluor 555, ab150114, Abcam), diluted in BSA 1% in PBS for 30 min at RT. For the detection of iM in cells, we adapted the protocol published in [10]. Birefly, cells were pre-fixed by adding directly to the media an equivolume of PFA 2% diluted in PBS 1×, incubated for 2 min at RT. Cells were washed with ice-cold PBS 1× and fixed with PFA 2% for 30 min at 4°C. Cells were then washed with ice cold PBS 1× and permeabilized using Triton X-100 0.1% in PBS 1× for 30 min at 4°C. Cells were blocked with iMab blocking buffer (BSA 2%, milk 1% in PBS 1×) o/n at 4°C. Slides were incubated with the primary antibody (iMab, 1:100, Ab01462-23.0, Absolute Antibody) o/n at 4°C. Finally, secondary antibody staining was performed (Alexa Fluor 488, 111-545-003, Jackson ImmunoResearch; Alexa Fluor 633, A211050, A-21071, Invitrogen; Alexa Fluor 555, ab150114, Abcam), diluted in PBS 1× supplemented wih 2% gelatin, 0.5% BSA, and 0.1% Tween-20 for 45 min at RT. After each antibody incubation, cells were washed three times with ice-cold PBS 1×, complemented with 0.1% Tween-20. For the proximity ligation assay (PLA), the kit Duolink Proximity Ligation Assay (DUO92007, Merck) was used, following the manufacturer’s instructions. Briefly, following the incubation with primary antibodies, cells were incubated with PLA probes for 1 h at 37°C (PLA probe anti-mouse minus, DUO92004; PLA probe anti-rabbit minus, DUO92002, Merck). Afterwards, slides were incubated for 30 min at 37°C with the ligation solution, which was followed by the incubation with the amplification solution for 100 min at 37°C. To detect telomeres, telomeric fluorescence *in situ* hybridization was performed using telomeric PNA probe TelG (PNABio, Cat No. F1008). After PLA staining, cells were fixed again with PFA 4% for 10 min at RT and then dehydrated through 2-min serial incubations with 70%, 85%, and 100% cold ethanol, followed by air-drying. TelG probe was diluted 1:100 in PNA FISH Hybridization Buffer (PNABio, Cat No. PFB01) complemented with Blocking buffer (PNABio, Cat. No. PFB05) and pre-heated, along with the slide, at 85°C for 5 min. The slide was incubated with the mixture for 10 min at 85°C to then complete hybrization at RT for 60 min. Slides were washed twice with wash solution (2× SSC, 0.1% Tween-20) at 55°C for 10 min, and once at RT. For each protocol, nuclei were stained and sildes mounted with mounting medium containing DAPI (Fluoroshield with DAPI – F6057, Merck). Images were acquired with a confocal microscope (Leica TCS SP8, Leica Microsystems).

### Telomere restriction fragment assay

Telomere length analysis was performed by using TeloTAGGG Telomere Length Assay Kit (12209136001, Merck) which exploits the telomere restriction fragment (TRF) principle. Briefly, genomic DNA was isolated from U2OS and HeLa cells using the QiAmp DNA Mini kit (51304, Qiagen). DNA from U2OS was sonicated on Bioruptor Plus (Diagenode) at maximum power for four times for 30 s, with breaks of 30 s. DNA was digested with both Hinf I and Rsa I endonuclease enzymes for 2 h at 37°C. After digestion, samples were loaded on a 0.8% agarose gel and run for 4 h. The gel was incubated with an 0.25 M HCl solution for 10 min, followed by two consequent incubations with an alkalaline denaturation solution (0.5 M NaOH, 1.5 M NaCl). Afterwards, the gel was incubated twice with a neutralization solution (0.5 M Tris–HCl, 3 M NaCl, pH 7.5) for 10 min, blotted o/n by capillary transfer on a nylon membrane (HybondN + Boehringer Mannheim), and UV-crosslinked (120 mJ). Lastly, DNA on the membrane was detected by hybridization with a digoxigenin-labeled telomeric probe, according to the manufacturer’s instructions. The membrane was scanned using a Molecular Imager Chemidoc XRS scanner (Bio-Rad) with ImageLab^TM^ software (Bio-Rad). The profiles and the length of fragments were quantified using the WALTER web tool [41].

### Statistical analysis

The results are presented as means ± SD, and data analysis was performed with the Prism GraphPad 7.0 software. For comparison between two groups, Student *t*-test was used. For comparisons between multiple groups, ordinary one-way ANOVA was used. In all tests, a *P*-value < .05 is symbolized by a single asterisk (*), while a *P*-value < .01 is symbolized by two asterisks (**), < .001 by three asterisks (***), and < .0001 by four asterisks (****).

## Results

### Biophysical characterization of telomeric iM

**Figure 1.**
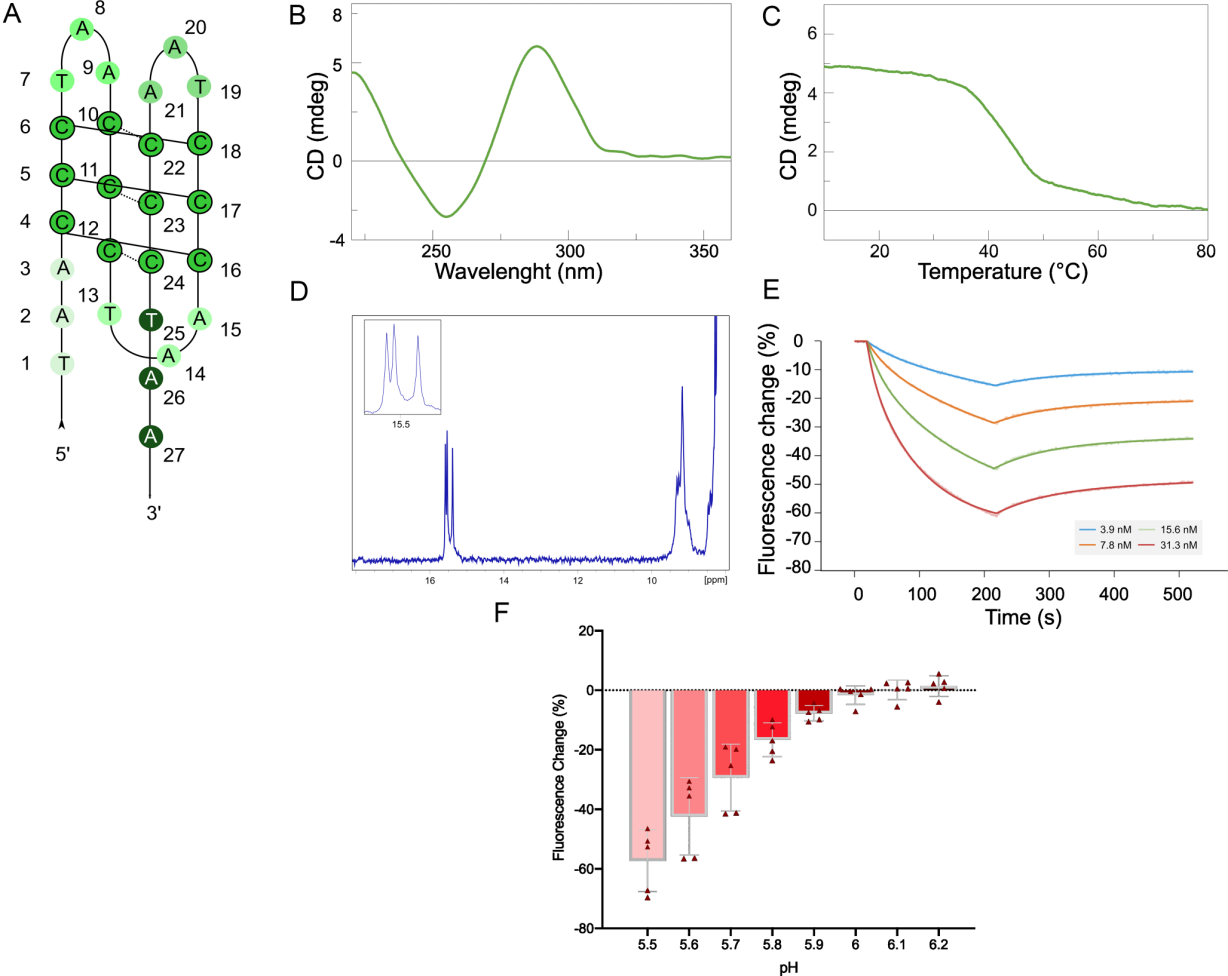
Biophysical characterization of the telomeric iM. (**A**) Schematic representation of the iM-containing C-NAT. Numbers indicate each nucleotide position in the ODN. The cytosine core is monochromatically colored and hydrogen bonds are represented by a line between cytosines. (**B**) CD spectrum of the C-NAT. On the *x*-axis, the wavelength is reported (nm), while on the *y*-axis, the CD value (mdeg). (**C**) CD melting profile of the C-NAT. Temperature (expressed in°C) and CD (expressed in mdeg) are reported on the *x*- and *y*-axis, respectively. (**D**) 1D ^1^H NMR spectrum of the C-NAT. (**E**) Real-time fluorescence signals and fits of a representative experiment measuring the association and dissociation phases of iMab at stated concentrations toward immobilized C-NAT at pH 6.5. Time (expressed in s) and Fluorescence change (expressed in %) are reported on the *x*- and *y*- axis, respectively. (**F**) Histogram showing the fluorescence change of C-NAT dye environment (expressed in %, *y*-axis) caused by each different pH (*x*-axis), calculated in the plateau phase of five different replicates.

In order to perform binding studies of APE1 toward telomeric iM structure, we checked the ability of a synthetic 27-mer oligonucleotide to fold into an iM structure, through different biophysical techniques and in buffers at different pHs. This oligonucleotide, named C-NAT, holds the nucleotide composition of the cytosine-rich strand of telomeres (Fig. 1A and Table 1). First, CD analysis at pH 5.5 revealed that C-NAT exhibits a positive peak at 288 nm and a negative one at 255 nm (Fig. 1B), typical of iM structures [42, 43], with a melting temperature (*T*_m_) of 46°C (Fig. 1C). The folding at pH 5.5 was confirmed by 1D ^1^H NMR spectroscopy (Fig. 1D), in which only three characteristic and unique imino sharp-resonance peaks (15–16 ppm) can be observed, in a pattern corresponding to two symmetry-related C:C^+^ pairs, indicating the presence of intercalated, hemiprotonated cytosine:cytosine base pairs in a single stable conformation. Then, we used the SwitchSENSE technology, by means of two different approaches, to further validate the iM folding (Supplementary Fig. S1A and C. Briefly, the first approach relies on the iM recognition by the iMab antibody (Supplementary Fig. S1A), able to specifically recognize iM structures both *in vitro* and in cells [10]. Keeping the C-NAT concentration constant and increasing iMab concentrations, we obtained dose-response profiles both at pH 5.5 and 6.5. Whereas iMab could not dissociate from the iM at pH 5.5, resulting in a non-measurable dissociation constant (*K*_D_) (Supplementary Fig. S1B), at pH 6.5 the fitting of the kinetic curves in a biphasic model allowed to determine the first (*K*_D1|1_) and the second dissociation constant (*K*_D2|2_) of the iMab toward the iM structure, which resulted of 9.01 nM for the *K*_D1|1_ and of 0.70 nM for the *K*_D2|2_ (Fig. 1E). For the second approach, the C-NAT was modified to hold the BBQ650 (Supplementary Fig. S1C). By increasing the pH of the buffer from 5.5 to 6.2, the signal of the Ra-dye attached to the adaptor sequence was quenched far less due to the unfolding of the iM structure which leads the BBQ increasingly further from the dye (Supplementary Fig. S1D). The change in fluorescence signal (%) decreased in a linear way upon 0.1 pH changes, confirming the importance of the acidic pH for the structure folding *in vitro* (Fig. 1F) and demonstrating that the complete unfolding of the C-NAT is reached at pH 6. All these experiments clearly confirmed that C-NAT is able to fold into an iM structure.

### APE1 is able to stably bind iM telomeric sequences

**Figure 2.**
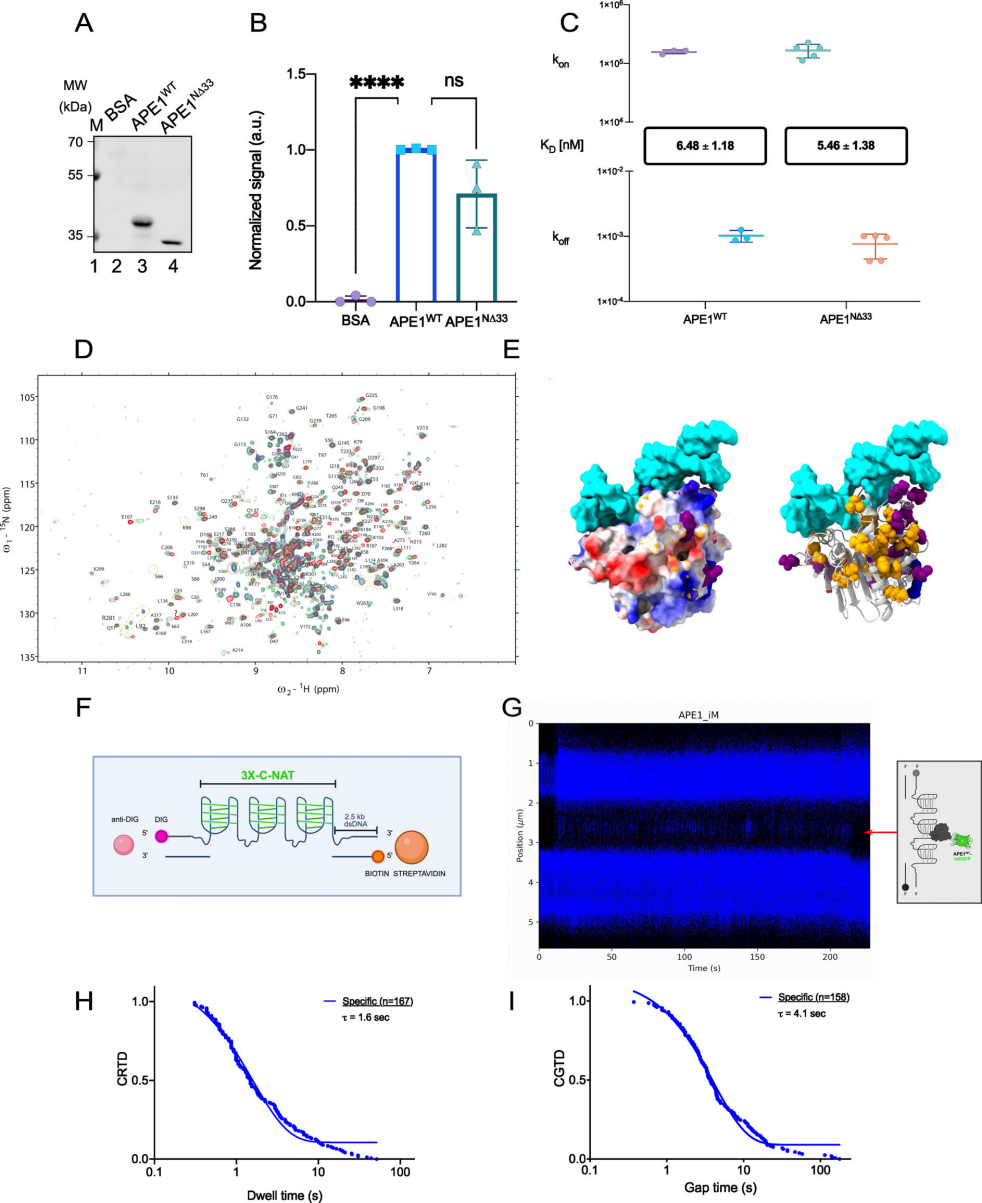
APE1 binds iM telomeric structures. (**A**) Representative SWB assay between the C-NAT and recombinant APE1^WT^ and APE1^N∆33^ proteins. BSA was used as a negative control. On the left, the electrophoretic marker is loaded, and the different molecular weights are expressed in kDa. (**B**) Relative histogram summarizing the different binding abilities of three independent replicates toward C-NAT between BSA, APE1^WT^, and APE1^N∆33^, normalized on the respective total protein staining signal (Supplementary Fig. S2A). (**C**) Graph representing the summary of C-NAT-APE1^WT^ and C-NAT-APE1^N∆33^ kinetic parameters (*k*_on_ and *k*_off_), expressed in nM, in MES buffer pH 5.5. *K*_D_ values, expressed in nM, are reported (*n* = 3). (**D**) Representation of 2D {^15^N-^1^H} TROESY-HSQC NMR spectra recorded at 25°C. Measurements were performed with APE1^WT^ alone (red), 0.6 (green), and 1:1 (blue) molar ratios of C-NAT to APE1^WT^. (**E**) Depiction of NMR-derived binding analysis on APE1 structure (PDB code: 1bix). Cyan represents deposited canonical duplex DNA (for reference only). Purple represents residues whose chemical shift variations (Δδ), before and after inclusion of i-motif DNA, are equal or superior to 2 standard deviations from the mean. Orange represents residues that lost over 2/3 or the respective peak volume. (**F**) Schematic representation of the three consecutive telomeric iM (3×-C-NAT) embedded in a DNA sequence used for the single-molecule analysis. (**G**) Representative kymograph of APE1^WT^-mEGFP from A549 clone 31 NCE binding to the iM (3×-C-NAT) substrate. Time (expressed in s) and position (expressed in µm) are reported on the *x*- and *y*-axis, respectively. The iM position is indicated with the red arrow. (**H**) CRTD plot of specific binding events of APE1^WT^-mEGFP to 3×-C-NAT. (**I**) CGTD plot of APE1^WT^-mEGFP association with 3×-C-NAT under the tested conditions.

Then we investigated APE1 ability to bind C-NAT *in vitro*. As a first qualitative approach, we performed a SWB assay by analyzing the binding ability of equal amounts of recombinant purified APE1 wild-type (APE1^WT^) and its mutant lacking the first 33 residues (APE1^N∆33^) (Fig. 2A and B; Supplementary Fig. S2A). The binding of both proteins toward C-NAT (Fig. 2A, lanes 3 and 4) was observed and resulted specific, since the inability to bind exhibited by BSA protein (Fig. 2A, lane 2). To deepen insights into any potential differences in binding between APE1^WT^ and APE1^N∆33^, we employed the SwitchSENSE technology by functionalizing the chip surface with C-NAT and modulating the ligand concentration of both recombinant proteins at pH 5.5. By fitting the dose-response curves in a monophasic model, we obtained similar *K*_D_ for the two proteins, respectively of 6.48 nM (±1.18) for APE1^WT^ and of 5.46 nM (±1.38) for APE1^N∆33^, demonstrating that both proteins show high affinity toward C-NAT with a minimal impact of the N-terminal region of APE1 in the binding affinity (Fig. 2C and Supplementary Fig. S2B and C. Additionally, 2D NMR spectroscopy analysis showed that the interaction between the C-NAT and APE1^WT^ caused the reduction of some peak intensities and chemical shift variations (Δδ) (Fig. 2D). The progressive decrease of peak intensities from the free protein (red) upon increasing concentrations of C-NAT (green and blue) suggested that the binding occurs in the intermediate exchange regime on the NMR timescale. The greater reduction in peak intensities at a 1:1 ratio (blue spectrum) suggested that a higher fraction of APE1^WT^ is engaged in binding, likely reaching near saturation. At a 1:1 molar ratio, the binding APE1^WT^ to the C-NAT does not substantially alter the core structure, as evidenced by the preservation of the characteristic imino proton peaks near 15.5 ppm. Moreover, as we can observe from Fig. 2E and Supplementary Fig. S2D, the C-NAT bound preferably to a distinct region of the canonical duplex DNA binding. The residues most involved in the binding are concentrated within and around α-helices 7 and 9. These helices are within the APE1 catalytic domain and contribute to DNA binding and stabilization of the enzyme–substrate complex. Interestingly, all these experiments were performed at pH 5.5, which contributed to Histidine (His) protonation and the increase of basic surfaces recognized by nucleic acids. In total, four His are within this new basic surface: H151 and H215 underwent changes, and H255 and 289 (blue) were not unambiguously identified in the 2D NMR spectra.

To characterize the binding of APE1 to the telomeric iM structure in a more physiological context, we performed a SMADNE with the C-Trap^®^ technology [39]. To perform this experiment, we designed a DNA sequence containing three consecutive telomeric iMs (called 3×-C-NAT), ligated in the middle of two 2.5 kb dsDNA tracts on each side, called DNA handles, derivatized at their extremities with three biotins or three digoxigenins, respectively (Fig. 2F). To set up the assay in the microfluidic system, we modulated the distance between the two beads, at pH 5.5, and detected the efficient formation of iM structures, which unfolded at an average force of 22 pN as evident from the force–distance curves of the tethered DNA (Supplementary Fig. S2E). Then, we performed the SMADNE analysis by monitoring the iM binding of single mEGFP-labeled APE1 proteins in the context of the nuclear cell extract. Specifically, we used NCE obtained from the A549 clone 31 cell line, endogenously expressing APE1–mEGFP and deeply characterized in Supplementary Fig. S3. To visualize and assess APE1 binding to iMs, 3×-C-NAT DNA was kept at a force of 15 pN and exposed to the NCE (Fig. 2G). Importantly, for the first time we were able to directly visualize APE1 engaging with iM, observing numerous specific binding events of APE1–mEGFP to the iM. Indeed, the duration of the iM binding events had an average lifetime ($\tau $) of 1.6 s (Fig. 2H) with 4.1-s gap times between the binding events (Fig. 2I). Taken together, these findings clearly confirmed the ability of APE1 to specifically bind the C-NAT sequence and suggested that the unstructured N-terminal region does not affect the overall affinity of the complex but rather plays a significant role in stabilizing the DNA–protein complex formation.

### The position of AP-sites in the iM influences its stability and APE1 binding

**Figure 3.**
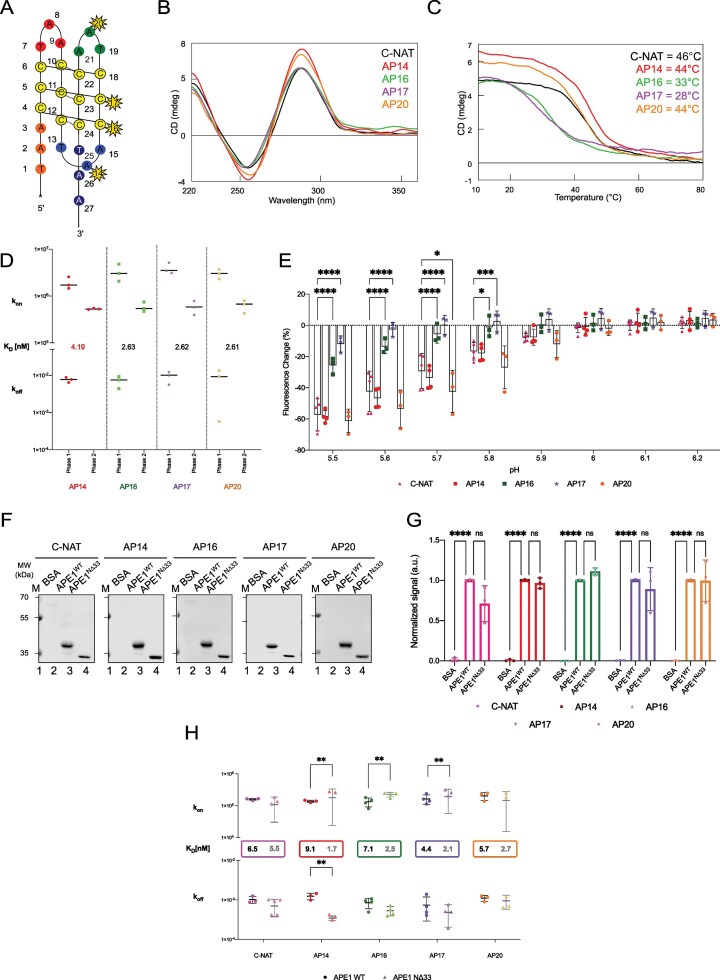
iM are structurally affected when containing AP-sites and are specifically bound by APE1. (**A**) Scheme of the damaged telomeric iM ODNs, holding an abasic site at different positions (AP14, AP16, AP17, AP20), indicated by a star. (**B**) CD spectrum of damaged ODNs. On the x-axis, the wavelength is reported (nm), while on the y-axis the CD value (mdeg). (**C**) CD melting profile of damaged ODNs. Temperature (expressed in°C) and CD (expressed in mdeg) are reported on the x- and y- axis, respectively. (**D**) Graph representing the summary of iMab kinetic parameters, including K_on_ and K_off_, toward each of the ODNs examined in MES buffer pH 6.5. K_D_ values, expressed in nM, are reported (n = 3). (**E**) Histogram showing fluorescence change of the different ODNs dye environment (expressed in %, y-axis) caused by increasing pH (x-axis), calculated in the plateau phase of five different replicates. (**F**) Representative SWB assays between each ODNs, as indicated upon each panel, and APE1^WT^ or APE1^N∆33^ proteins. BSA was used as a negative control. On the left, the electrophoretic marker is loaded, and the different molecular weights are expressed in kDa. (**G**) Relative histogram summarizing the different binding abilities of three independent replicates toward the ODNs used in this study between BSA, APE1^WT^, APE1^N∆33^, normalized on the respective total protein staining signal (Supplementary Fig. S4C). (**H**) Graph representing the summary of APE1^WT^ and APE1^N∆33^ kinetic parameters, including k_on_ and k_off_, toward each of the ODNs examined in MES buffer pH 5.5. K_D_ values, expressed in nM, are reported (n = 3).

APE1 repair efficiency of AP-sites embedded in iM tetraplex structures is unknown and requires investigation. We aimed to characterize the cleavage activity of APE1 on damaged iM by evaluating its endonuclease activity on AP-sites placed at different positions in the C-NAT sequence. Specifically, we designed: AP14 and AP20 ODNs bearing an AP-site in the second and in the third loop, respectively, both substituting an adenine base; AP16 and AP17 ODNs holding the AP-site in the core of the iM structure, substituting the first and middle cytosine of the third C-run, respectively (Fig. 3A and Table 1).

First, we assessed the impact of AP-sites on the formation and stability of the iM itself through CD spectroscopy. As shown in Fig. 3B, all the AP-site-containing sequences showed CD profiles similar to that of the C-NAT with a positive band at 288 nm and a negative one at 255 nm (Fig. 1C). Specifically, AP14 and AP20 had a T_m_ of 44°C, comparable to the C-NAT (46°C), while AP16 and AP17 of 33°C and 28°C, respectively (Fig. 3C), suggesting that iMs formed by AP16 and AP17, which present AP-sites in the C-core, are much less stable with respect to those having these sites in the loops, AP14 and AP20. We also used the two SwitchSENSE approaches, previously described, to further analyze potential differences in the iM folding ability among the damaged sequences. We first validated the iM folding with the iMab antibody at pH 6.5 (Fig. 3D and Supplementary Fig. S4A). The best-suited fitting algorithm was the biphasic model, in which only the first dissociation constant (*K*_D1|1_) of the iMab toward the different ODNs was calculable. Despite the different positions of AP-sites within the iM, iMab bound each ODN with dissociation constants in the low nanomolar range, with minor differences between them. Furthermore, by using the second approach (Fig. 3E and Supplementary Fig. S4B), we found that AP14 and AP20 have a similar behavior to the C-NAT, showing parallel levels of fluorescence change (%), whereas AP16 and AP17 behave in a significantly different way since, already at pH 5.5, they showed a 35% difference in fluorescence change compared to the undamaged C-NAT, indicating that a lower percentage of the total amount of these ODNs was folded compared to the undamaged one. Moreover, in agreement with the *T*_m_ values, we observed a difference between AP16 and AP17, as AP16 seemed to be slightly more stable than AP17. In summary, whereas C-NAT, AP14, and AP20 were stable until reaching pH 6.0, AP16 and AP17 unfolded at pH 5.8 and 5.7, respectively, thus confirming that the position of the AP-site crucially influences iM structure.

Subsequently, the ability of APE1 to recognize damaged telomeric iM structures was investigated *in vitro*: in the SWB assay, by using equal amounts of APE1^WT^ and APE1^N∆33^ (Supplementary Fig. S4C), both proteins bound modified iM with not statistically significant differences (Fig. 3F and G). To complement the qualitative data obtained by SWB, the SwitchSENSE assay was employed next. By fitting the dose response curves with a monophasic model, we obtained *K*_D_ values in the nanomolar range for both proteins for each ODN. In detail, APE1^WT^ bound the ODNs with similar affinities (Fig. 3H and Supplementary Fig. S4D), while APE1^N∆33^ exhibited higher affinity toward the damaged ODNs compared to the C-NAT (Fig. 3H and Supplementary Fig. S4E) and also in comparison with APE1^WT^. To further characterize the contribution of the N-terminal region of APE1 to the formation of the iM complex, we employed a UV-crosslinking coupled to SDS–PAGE analysis (Supplementary Fig. S4F). We observed that APE1 was able to bind each ODN, forming a stable complex of 50 kDa (as indicated by a single asterisk), while for APE1^N∆33^ only weak bands were detected, as indicated by a double asterisk. These results suggest that the N-terminal region contributes to stabilize the protein–DNA complex once formed, rather than affect the overall binding affinity. Notably, a similar reduction in crosslinking signal for APE1^NΔ33^ was also observed on natural ss (ss_dF) and ds (ds_dF) control ODNs (Supplementary Fig. S4G), suggesting that this effect may partly reflect the loss of lysine residues in the N-terminal region, which could contribute to UV-crosslinking efficiency independently of DNA binding.

### APE1 endonuclease activity on iM strongly depends on AP-site position and on its unstructured N-terminal region

**Figure 4.**
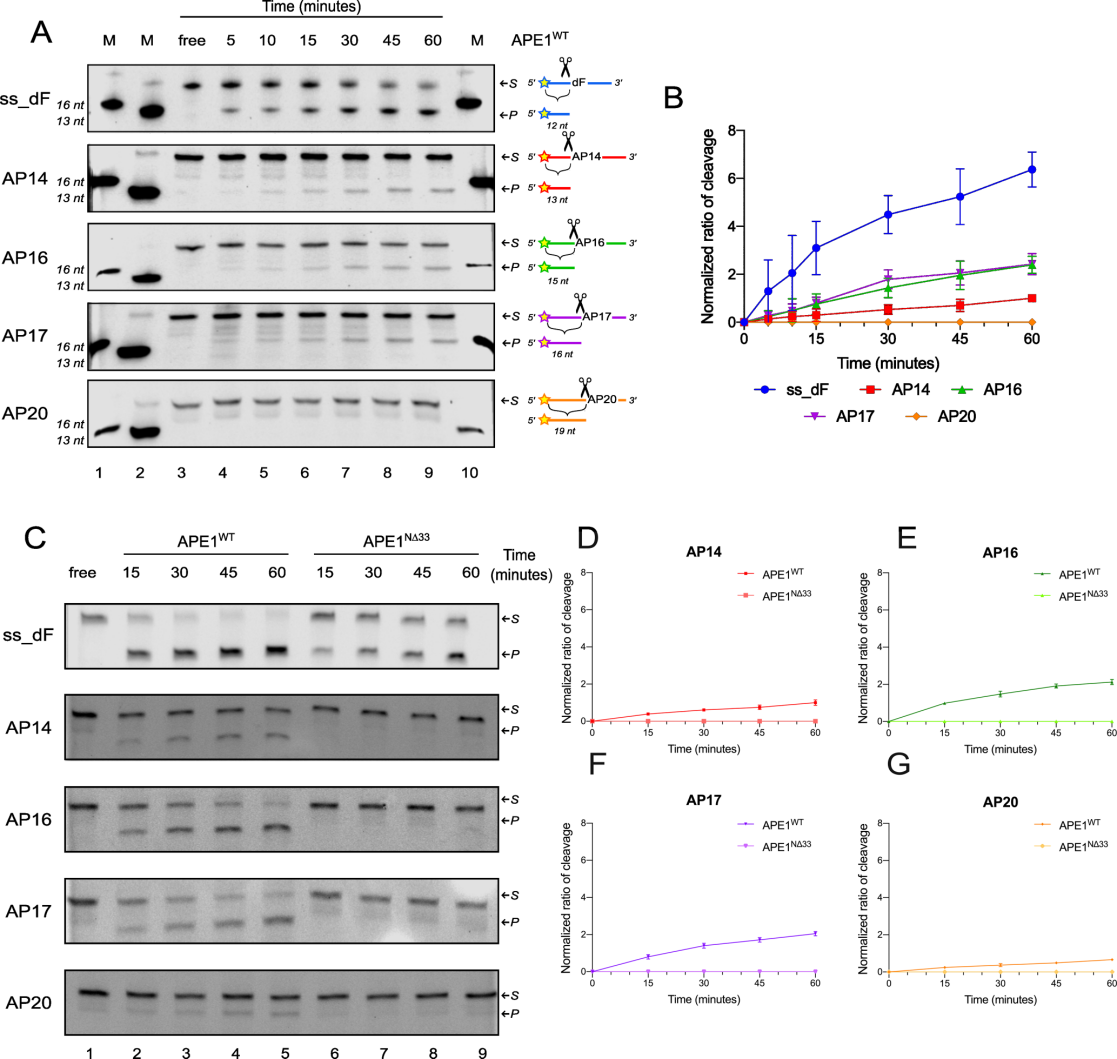
The position of the AP-site on the telomeric iM influences APE1 endonuclease activity, which relies on its N-terminal region. (**A**) Representative denaturing polyacrylamide gels of time-course kinetics APE1^WT^ cleavage activity. Lanes 1 and 2 indicate DNA fragments with known length (16 and 13 nt, respectively). The “free” sample represents the control without protein (lane 3). On the right, the substrate and the product bands are indicated by two arrows, and the expected length of the cleavage product is indicated upon the AP-site. A constant dose of APE1 (80 nM) was incubated with the respective oligonucleotide at 37°C. (**B**) Relative graph depicting the time (expressed in minutes) and normalized ratio of cleavage (considering the cleavage of AP14 at 60 min equal to one) on the *x*- and *y*-axis, respectively. Data are expressed as mean ± SD of three independent technical replicates. (**C**) Representative denaturing polyacrylamide gels of APE1^WT^ (lane 2–5) and APE1^N∆33^ (lane 6–9) cleavage activity performed on all substrates (lane 2–9). The “free” sample represents the control without protein (lane 1). On the right, the substrate and the product bands are indicated by two arrows. A constant dose of APE1^WT^ or APE1^N∆33^ (120 nM) was incubated with the respective oligonucleotide at 37°C, and the reactions were stopped at different time points, indicated on the gel. Relative graphs illustrating the time-course kinetics activity of APE1^WT^ and APE1^N∆33^ recombinant proteins on AP14 (**D**), AP16 (**E**), AP17 (**F**), and AP20 (**G**). Time (expressed in minutes) and normalized ratio of cleavage (considering the cleavage of AP14 at 60 min equal to one) are reported on the *x*- and *y*-axes, respectively. Data are expressed as mean ± SD of three independent technical replicates.

To test if the AP-site position on the telomeric iM structure might influence APE1 endonuclease activity, we performed an AP-site incision assay (Fig. 4A). As a positive control of APE1^WT^ activity, we used a ss oligonucleotide (ss_dF, Table 1) designed to lack any secondary structure formation ability. Among the iM-forming substrates, AP16 and AP17 were preferentially cleaved with respect to AP14 and AP20, while AP20 was not cleaved under these conditions (Fig. 4B). Normalizing these data to AP14 cleavage at 60 min, the different substrates were processed in the following order: ss_dF (6.3) > AP17 (2.42) > AP16 (2.39) > AP14 [1] > AP20 (0). These results point out that APE1^WT^ cleaves more efficiently damaged iM bearing the AP-site in the core of the structure rather than in the loops, and that these differences could be connected to the ability of AP14 and AP20 to conserve iM folding with greater stability, which presumably inhibits APE1^WT^ endonuclease activity. To confirm that the observed cleavage activity on iM substrates reflects genuine APE1 endonuclease activity rather than stochastic iM unfolding, we tested two well-characterized, catalytically dead mutants, APE1^H309N^ and APE1^E96A^ (Supplementary Fig. S5A), alongside treatment with the APE1 enzymatic inhibitor compound 3 (#3) [44] (Supplementary Fig. S5B). Neither catalytic mutant nor the inhibitor-treated WT protein produced any detectable cleavage on any substrate tested, including ss_dF, ds_dF, and all four iM-forming substrates (AP14, AP16, AP17, AP20), whereas APE1^WT^ cleaved all substrates as expected (Supplementary Fig. S5A and B. These data confirm unambiguously that the cleavage observed in our assays is a direct consequence of APE1 catalytic activity. To better investigate the differential cleavage among core- and loop-damaged iMs, we performed the cleavage assay by using the ODNs in a regular Watson–Crick ds conformation in order to avoid iM structure folding (Table 1). As shown in Supplementary Fig. S5D, APE1^WT^ was able to cleave each ds_ODN at a lower concentration than the ss_ODN counterpart (0.125 nM versus 80 nM, respectively) and more rapidly (15 min versus 60 min, respectively). Indeed, at 15 min, both ds_AP14 and ds_AP16 reached a $ \sim $50% of cleavage, while both ds_AP17 and ds_AP20 reached the $ \sim $30% (Supplementary Fig. S5D–H), corroborating previous data showing that AP-sites located toward the 3′ were processed slightly less efficiently in the dsODNs, but at the same time confirming a major effect of the AP-site position within the iM structure.

In order to evaluate the impact of the absence of the N-terminal region of the protein on its catalytic activity on damaged iM, we performed similar experiments by employing APE1^N∆33^ and surprisingly found that APE1^N∆33^ did not cleave any substrate (Fig. 4C–G). To dissect whether this effect reflects a general loss of endonuclease activity or a specific requirement for non-canonical structural contexts, we performed cleavage assays on the unstructured ss_dF (Fig. 4C and Supplementary Fig. S5C). Both APE1^WT^ and APE1^NΔ33^ retained enzymatic activity toward this substrate, although APE1^NΔ33^ displayed ~35% lower cleavage efficiency at each time point examined. As control, the cleavage assay with ds_ODNs was carried out (Supplementary Fig. S5D–H); in this case APE1^WT^ and APE1^N∆33^ processed each ds_ODN to a comparable extent, as already reported [45]. In addition, the presence of APE1^WT^ can induce conformational changes in the iM fold of AP20 at a superstoichiometric ratio, consistent with local or partial destabilization of the iM structure (Supplementary Fig. S5I). Collectively, these results demonstrate that the N-terminal region of APE1 is not strictly required for endonuclease activity on canonical substrates, but becomes essential when the protein acts on lesions embedded within non-canonical DNA secondary structures such as G4s and iMs, likely by facilitating productive enzyme-substrate engagement through stabilization of the protein–DNA complex.

### APE1 enzymatic activity on damaged telomeric iM is inhibited by poly(rC)-binding protein 1

**Figure 5.**
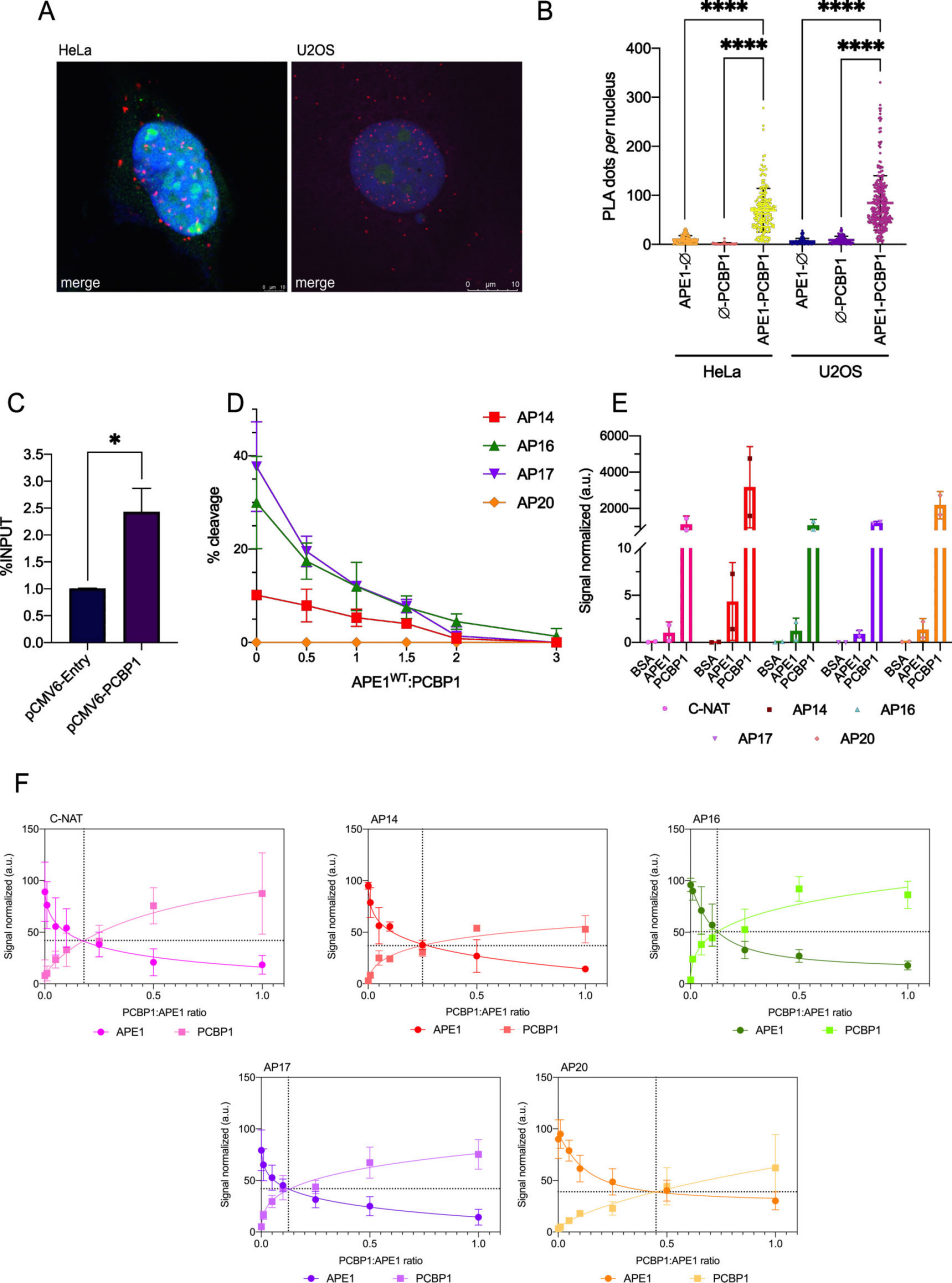
Damaged telomeric iM cleavage mediated by APE1 is modulated by APE1 interaction with PCBP1. (**A**) PLA analysis of the interaction between APE1 and PCBP1 proteins in HeLa and U2OS cells (PLA dots in red). The merged panel shows the overlay between the four channels, including APE1 and PCBP1 staining in green (rabbit-488) and magenta (mouse-633), respectively, and the nuclear DAPI staining in blue. The scale is indicated and expressed in μm. (**B**) Graph depicting the number of PLA dots per nucleus in U2OS and HeLa cell lines. Average and standard deviation values are plotted (*n* = 3). (**C**) qPCR validation of PCBP1 binding toward telomeric sequences in U2OS cells transfected with pCMV6-ENTRY and pCMV6-PCBP1 plasmids by telo-ChIP analysis. Data are represented as fold change percentage of the amount of immunoprecipitated target on the amount of the target present in the total input RNA (% INPUT) and normalized to pCMV6-ENTRY. Data are expressed as means ± SD of two independent biological replicas. (**D**) Relative graph illustrating the activity of APE1^WT^ recombinant protein after pre-incubation with different amounts of PCBP1 on AP14, AP16, AP17, and AP20. PCBP1 concentration (nM) and percentage of cleavage (%) are reported on the *x*- and *y*-axis, respectively. Data are expressed as mean ± SD of three independent technical replicates. (**E**) Relative histogram summarizing the different binding abilities toward each ODN between BSA, APE1^WT^, and PCBP1 (*n* = 3), normalized on the respective total protein staining signal. (**F**) Graphs showing the signal intensity of APE1 or PCBP1 proteins normalized to their signal when incubated alone with the substrate (expressed in a.u., *y*-axis) against the PCBP1:APE1 ratio (*x*-axis). Sigmoidal curves were fitted to the resulting values (*n* = 3).

In our previous studies on APE1 interactome in HeLa cells [46], we found several hnRNPs that are involved in modulating iM folding, including PCBP1 [23]. Indeed, PCBP1 binds to several iM, maintains their folding, and plays a role in regulating the competing formation of iM and G4 in the genome of human cells [23]. Therefore, we better characterized the close proximity (<40 nm) between APE1 and PCBP1 in our telomeric model, using PLA analysis. By using PLA analysis, the occurrence of APE1–PCBP1 interaction in HeLa cells was confirmed (Fig. 5A and Supplementary Fig. S6A) and evaluated also in U2OS cells from osteosarcoma (Fig. 5A and Supplementary Fig. S6B). APE1 specifically interacted with PCBP1, in both nuclear and cytosolic compartments, with a higher number of PLA dots in the nucleus, with a mean of 70 dots per nucleus in HeLa and 84 dots per nucleus in U2OS (Fig. 5B). These data were also confirmed by PLA analysis on HeLa cells transiently silenced for APE1, PCBP1, or both proteins, leading to the loss of the interaction (Supplementary Fig. S6C), as well as by analyzing two stable APE1 knockout (KO) clones derived from U2OS cell line, called U2OS^19^ and U2OS^21^, which were transiently silenced for PCBP1 (Supplementary Fig. S6D). In both models, the deficiency of one or both proteins resulted in the loss of PLA dots, as expected.

To assess whether the APE1/PCBP1 interaction detected by PLA occurs specifically at telomeres, we investigated PCBP1 direct binding to telomeric sequences. APE1 occupancy at telomeres in U2OS cells has already been demonstrated by telo-ChIP in previous studies [28, 47]. To do so, we performed a telo-ChIP assay in U2OS cells transfected for 48 h with either a control plasmid (pCMV6-ENTRY) or a plasmid driving expression of Flag-tagged PCBP1 (pCMV6-PCBP1) (Supplementary Fig. S6E). Following Flag immunoprecipitation, input and immunoprecipitated DNA fractions were subjected to qPCR analysis using telomere-specific primers. As shown in Fig. 5C, telomeric sequences were significantly enriched in the Flag-immunoprecipitated fraction from pCMV6-PCBP1-transfected cells compared to the control, demonstrating that PCBP1 associates with telomeric chromatin in U2OS cells. To provide orthogonal spatial evidence of APE1/PCBP1 interaction at telomeres, we performed PLA combined with telomere-FISH in U2OS cells. As shown in Supplementary Fig. S6F, a subset of PLA signals (in red), reflecting sites of close proximity between APE1 and PCBP1, co-localized with FISH signals (in green) marking telomeric sequences. Importantly, the partial rather than complete overlap between PLA and FISH dots is fully consistent with the known biology of both proteins, given their ubiquitous expression and their multiple roles in genome maintenance beyond telomere biology. Together with the previously published APE1 telo-ChIP data [28, 47], these results provide direct evidence that both proteins reside at telomeres and strongly support our model in which APE1 and PCBP1 functionally interact at telomeric loci.

We next investigated whether PCBP1 exerts its role in maintaining genome integrity [23] by functionally modulating the enzymatic activity of APE1 on damaged iM structures. As expected, PCBP1 was not able to exert any endonuclease activity on damaged iMs alone (Supplementary Fig. S7A, lanes 4). Interestingly, the pre-incubation of damaged iM with PCBP1 and the subsequent addition of APE1^WT^ had a strong dose-dependent inhibitory effect on APE1^WT^ endonuclease activity, commonly observed for any ODNs (Supplementary Fig. S7A, lanes from 5 to 9). Specifically, the pre-incubation with an under-stoichiometric dose of PCBP1 compared to APE1^WT^ (ratio: 0.5:1, lane 5) was already sufficient to inhibit the product formation of ∼50% for AP16 and AP17, while of ~23% in the case of AP14. As expected, AP20, which is not efficiently processed by APE1^WT^, was not influenced by PCBP1 presence (Fig. 5D). By performing the same experiment on the ds_ODNs (Supplementary Fig. S7B and C, we observed that the pre-incubation with PCBP1 did not influence the cleavage ability of APE1^WT^, confirming that the inhibitory effect is specific for the presence of iM structure. Lastly, to assess if the inhibitory effect of PCBP1 was due to a possible competition with APE1 for the iM, we performed a SWB assay by loading equal amounts of BSA (as negative control) and APE1^WT^, together with 40-fold lower amount of PCBP1 protein (Supplementary Fig. S7D). After signal normalization on total protein staining, we observed that the binding of PCBP1 was at least 1000-fold higher compared to that of APE1^WT^ for each ODN (Fig. 5E). To provide further direct evidence of the competitive relationship between the two proteins, we performed UV-crosslinking competition assays, in which a constant amount of APE1 (20 pmol) was co-incubated with increasing concentrations of PCBP1, up to a 1:1 molar ratio, across all substrates. As shown in Supplementary Fig. S7E, PCBP1 effectively competes with APE1 for binding to all substrates in a concentration-dependent manner. Indeed, the PCBP1 signal increases progressively while the APE1 signal diminishes correspondingly. After normalizing the signal intensity of each protein to its signal when incubated alone with the substrate, sigmoidal curves were fitted to the resulting values (Fig. 5F). The crossing point of the two curves, representing the protein ratio at which the normalized binding signals are equal, was determined for each substrate and is indicated in brackets: C-NAT (0.18), AP14 (0.25), AP16 (0.12), AP17 (0.12), and AP20 (0.45). Strikingly, for all substrates, PCBP1 reaches binding parity with APE1 at sub-equimolar concentrations, indicating that PCBP1 outcompetes APE1 for iM binding even when present at a lower molar ratio.

Overall, these data provide quantitative support to suggest that PCBP1 has a higher binding affinity for the iM telomeric compared to APE1. Moreover, PCBP1 could shield these structures, exerting a competitive effect on APE1, thus inhibiting its cleavage activity.

### APE1 and PCBP1 depletion impacts on telomere length and on the interaction with telomeric repeat-binding factor 2

**Figure 6.**
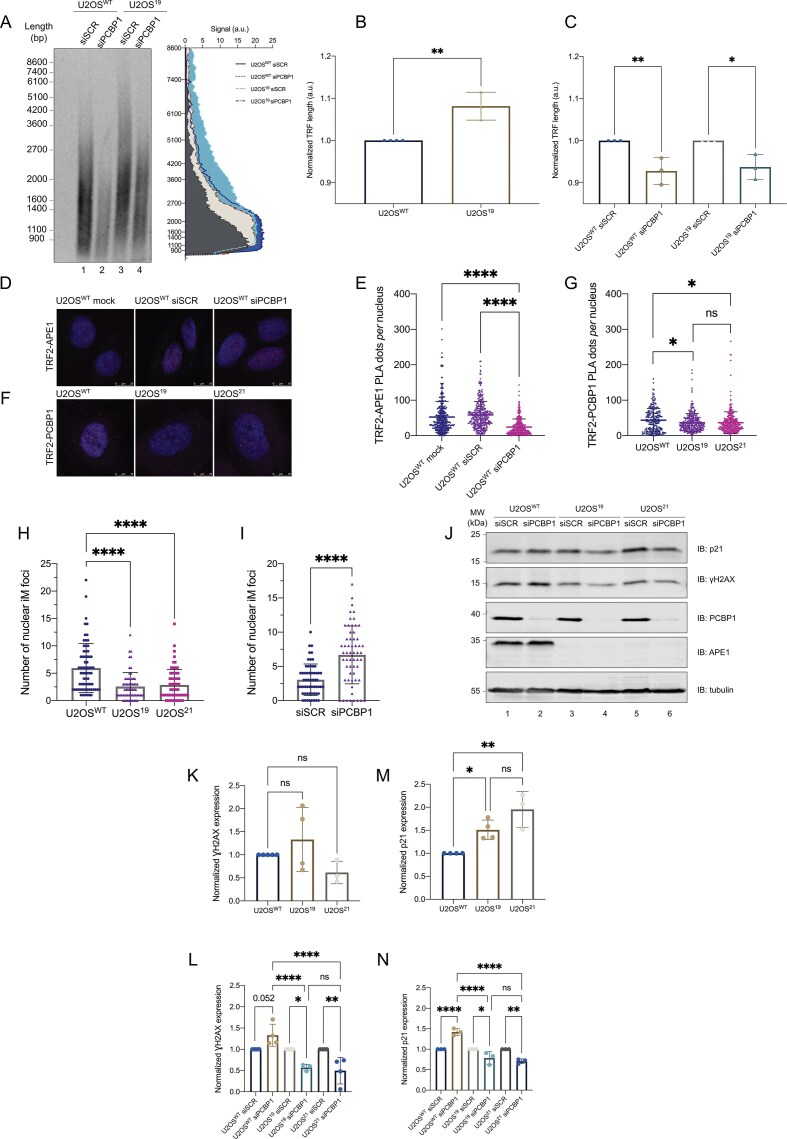
APE1 and PCBP1 depletion impacts on telomere length and on the interaction with TRF2 protein. (**A**) TRF assay was used to measure telomere length in U2OS cells expressing (U2OS^WT^) or KO for APE1 (U2OS^19^) protein, either silenced or not for PCBP1 protein (siPCBP1 and siSCR, respectively), as indicated upon the gel. On the left side of the gel, the molecular weight marker, as provided by the kit, is loaded, and the length of each band is indicated and expressed as bp. On the right side, a representative graph showing the intensity profiles relative to the TRF assay is reported. On the *y*-axis, the signal intensity is reported (expressed in a.u.), while on the *x*-axis, the TRF marker length (expressed in bp) is reported. (**B**) Graph reporting the normalized TRF mean length of U2OS^WT^ and U2OS^19^. TRF mean length is normalized to U2OS^WT^ values. Data are expressed as mean ± SD of three independent replicates. (**C**) Graph reporting the normalized TRF mean length of U2OS^WT^ and U2OS^19^ silenced for PCBP1. TRF mean length is normalized to each respective scramble silencing values. Data are expressed as mean ± SD of three independent replicates. (**D**) PLA analysis between TRF2 and APE1 proteins in U2OS cells, either silenced or not for PCBP1 protein (siPCBP1 and mock, siSCR, respectively). The representative merge panel shows PLA dots in red, TRF2 staining in green, and APE1 staining in magenta. The nuclear staining, obtained with DAPI, is in blue. The scale is indicated and expressed in μm. (**E**) Relative graph depicting the number of TRF2-APE1 PLA dots per nucleus in all U2OS^WT^ conditions. Average and standard deviation values are plotted (*n* = 3). (**F**) PLA analysis of the interaction between TRF2 and PCBP1 proteins in U2OS cells expressing (U2OS^WT^) or KO for APE1. The representative merge panel shows PLA dots in red, TRF2 staining in green, and and PCBP1 in magenta. The nuclear staining, obtained with DAPI, is in blue. The scale is indicated and expressed in μm. (**G**) Relative graph depicting the number of TRF2–PCBP1 PLA dots per nucleus in U2OS^WT^, U2OS^19^, and U2OS^21^ cell lines. Average and standard deviation values are plotted (*n* = 3). (**H**) Scatter plot of iMab analysis representing individual values of iM foci in the nuclear compartment of U2OS clones. Foci were counted for each cell, using DAPI as a nuclear mask. Data are expressed as means ± SD of two independent replicates. (**I**) Scatter plot of iMab analysis representing individual values of iM foci in the nuclear compartment of siSCR versus siPCBP1 conditions. Foci were counted for each cell, using DAPI as a nuclear mask. Data are expressed as means ± SD of two independent replicates. (**J**) Western blot analysis of p21, $\gamma $H2AX, PCBP1, and APE1 levels in U2OS^WT^, U2OS^19^, and U2OS^21^ cell lines, either silenced or not for PCBP1 (siPCBP1 and siSCR, respectively). Tubulin was used as normalizer. Molecular weights (MW, expressed in kDa) are reported on the left. (**K, L**) Densitometric analysis of $\gamma $H2AX expression levels, normalized to tubulin. In the upper panel (K), fold change values relative to U2OS^WT^, arbitrarily set to 1, are shown. In the lower panel (L), fold change values relative to each siSCR, arbitrarily set to 1, are shown. Values are mean ± SD of four independent replicates. (**M, N**) Densitometric analysis of p21 expression levels, normalized to tubulin. In the upper panel (M), fold change values relative to U2OS^WT^, arbitrarily set to 1, are shown. In the lower panel (N), fold change values relative to each siSCR, arbitrarily set to 1, are shown. Values are mean ± SD of four independent replicates.

To further explore the biological relevance of both APE1 and PCBP1 proteins, we examined their impact on telomere length maintenance by using U2OS cell line and their corresponding APE1-KO models. Due to the great length of U2OS telomeres, previous studies unsuccessfully tried to determine size changes upon transient APE1 silencing by using TRF assay [47]. In this context, we performed TRF assay (Fig. 6A) by sonicating the DNA obtained from U2OS^WT^ and U2OS^19^ cell lines, transiently silenced for PCBP1 (Supplementary Fig. S7A), before digesting with restriction enzymes (Supplementary Fig. S7B). Interestingly, APE1 KO led to a slight but significant increase in telomere length (Fig. 6B), whereas the transient silencing of PCBP1 in both WT and KO models led to the same slight but significant decrease of telomere length (Fig. 6C). The opposite behavior of these two proteins could be explained by their roles in maintaining telomere length at equilibrium when both proteins are present. To validate these findings and address potential confounding effects due to sonication, we performed complementary TRF analyses in HeLa cells, which carry shorter telomeres. Using a doxycycline-inducible APE1 KD system (HeLa SCR versus HeLa Cl.3) (Supplementary Fig. S8C), we observed consistent telomere shortening upon APE1 depletion across all conditions (Δ[SCR–Cl.3]: mock = 539 bp; siSCR = 314 bp; siPCBP1 = 269 bp), whereas PCBP1 silencing alone did not affect telomere length in this model significantly (Supplementary Fig. S8D and E. In a complementary transient co-silencing approach in standard HeLa cells (Supplementary Fig. S8F–H), APE1 depletion resulted in a 178 bp reduction in mean telomere length (siSCR: 3015 bp; siAPE1: 2837 bp), and PCBP1 silencing produced a modest but comparable reduction (Δ = 122 bp). Importantly, co-depletion of both proteins did not result in an additive effect (Δ = 118 bp). Taken together, the opposite effects of APE1 and PCBP1 observed in U2OS cells (Fig. 6B and C), and their convergent behavior in HeLa cells (Supplementary Fig. S8D–H), support a model in which the two proteins act in a coordinated manner to maintain telomere length homeostasis. The modest impact of PCBP1 loss in telomerase-positive HeLa cells likely reflects telomerase-mediated compensation, which is absent in the ALT context of U2OS cells, underscoring the complementary nature of the two experimental systems.

Previous studies have observed that both APE1 and PCBP1 could have a role in the maintenance of telomeres through the regulation of proteins belonging to the shelterin complex. Indeed, APE1 co-localizes with both TRF2 and POT1 proteins [47], whereas PCBP1 co-localizes with TRF2 and telomeric repeat-binding factor 1 (TRF1) [14]. TRF1 and TRF2 bind the ds region of telomeres and, specifically, TRF2 prevents the recognition of telomere ends as double-strand breaks (DSBs), inhibiting ataxia telangiectasia mutated (ATM)-activated DDR [48]. POT1 binds the G-rich overhang and protects it from cleavage [48]. The displacement of TRF2 and POT1 from telomeres is an important sign of telomere dysfunction [48]. It has been demonstrated that the depletion of APE1 leads to a decreased amount of TRF2 at telomeres, without affecting the overall amount of TRF2 in the cell [47]. To better study APE1 and PCBP1 role and their interplay at the telomere level, we examined their interaction with TRF2. Specifically, we first employed PLA analysis between APE1–TRF2 (Supplementary Fig. S9A and B and PCBP1–TRF2 (Supplementary Fig. S9C and D in U2OS^WT^ cells. Both APE1 and PCBP1 interacted with TRF2 in the nuclear compartment, with a mean of 53 dots per nucleus for APE1–TRF2 (Supplementary Fig. S9B) and of 43 dots per nucleus for PCBP1–TRF2 (Supplementary Fig. S9D). In order to assess the existence of any influence between APE1 and PCBP1 in the modulation of their interaction with TRF2, we transiently silenced PCBP1 in U2OS^WT^ cells, and we detected the presence of APE1–TRF2 PLA dots (Fig. 6D and Supplementary Fig. S9E). Although APE1 staining (in magent A) and TRF2 foci (in green) were both well-detectable after silencing PCBP1 without any effect, the PLA dots were far less apparent compared to the mock and siSCR conditions, with a dramatic reduction of ~60% compared to the siSCR condition (Fig. 6E). At the same time, we evaluated the interaction of PCBP1–TRF2 in both U2OS^19^ and U2OS^21^ APE1-KO cell models (Fig. 6F and Supplementary Fig. S9F). Again, although PCBP1 and TRF2 staining was not affected by the absence of APE1, the PLA dots upon APE1 KO were less and more faint compared to the WT, with a 16% reduction (Fig. 6G). In both PCBP1 silencing or APE1 KO conditions, the TRF2 expression levels evaluated by Western blot analysis didn’t show any significant downregulation (Supplementary Fig. S9G and H, confirming already previously published data [47].

Moreover, to further assess the interplay occurring between iM dynamics and APE1 in a cellular context, we explored whether APE1 depletion could influence the accumulation of iM foci in the nuclei of U2OS cells. With this aim, we performed an immunofluorescence analysis by using iMab antibody (Supplementary Fig. S9I). The signal appears as a single discrete foci that can be quantified. Surprisingly, a significant depletion of iMab dots of ~50% was observed in both APE1 KO clones U2OS^19^ and U2OS^21^, thus connecting APE1 to iM folding regulation also in cells (Fig. 6H). In contrast to the reduction of iMab-positive foci observed in APE1-KO clones, PCBP1 depletion in U2OS cells resulted in a significant increase in iMab nuclear foci (Supplementary Fig. S9J and Fig. 6I), consistent with the recently demonstrated role of PCBP1 as a selective iM-unfolding protein [49]. The opposite behavior of these two proteins supports a model in which APE1 and PCBP1 contribute to the regulation of iM folding.

Lastly, we focused our attention on how APE1 and PCBP1 depletion could influence the DDR pathway. Therefore, we analyzed the accumulation of DSB by measuring the activation of $\gamma $H2AX and a downstream target of the DDR and cell senescence, p21, which can be modulated at multiple levels by APE1 through its interplay with p53, upon depletion of either or both APE1 and PCBP1 proteins (Fig. 6J). APE1 depletion in U2OS^19^ and U2OS^21^ cell models did not result to a significant activation of $\gamma $H2AX compared to U2OS^WT^ under basal conditions (Fig. 6K). On the contrary, PCBP1 depletion led to ∼35% increase in phosphorylated $\gamma $H2AX (Fig. 6L), confirming previous results obtained in other cell lines [23]. Surprisingly, when both proteins were depleted, phosphorylated $\gamma $H2AX levels were even lower than in basal condition, specifically of the 40% for U2OS^19^ and 50% for U2OS^21^ compared to the respective siSCR (Fig. 6L), suggesting a possible impairment in the signaling of DNA damage when both proteins are absent. The levels of p21 protein after APE1 depletion in U2OS cell models resulted increased of the 50% in U2OS^19^ and 70% in U2OS^21^, confirming previous results linking APE1 depletion with p21 overexpression [50–52] (Fig. 6M). p21 levels were also higher upon PCBP1 silencing compared to siSCR, establishing that the absence of PCBP1 induces higher levels of DNA damage with downstream relevance to the activation of p21 (Fig. 6N). Lastly, we observed that p21 levels decreased when PCBP1 was silenced in U2OS^19^ and U2OS^21^, respectively, by the 22% in U2OS^19^ (compared to its siSCR) and of the 30% in U2OS^21^(compared to its siSCR), almost taking back p21 expression to its basal levels (Fig. 6N).

In summary, all these experiments allowed us to conclude that APE1 and PCBP1 contribute to telomere stability in ALT cells by maintaining telomere length at equilibrium when both present and by modulating the interaction of the other with shelterin protein TRF2.

## Discussion

The maintenance of genome stability at non-canonical DNA secondary structures represents an emerging and largely unexplored topic in DNA repair biology. While the interplay between BER proteins and G4 structures has been extensively characterized, the equivalent process occurring at iM level has been overlooked. AP-sites, the primary repair intermediates generated by DNA glycosylases, have been proposed to hold regulatory roles when embedded in G4-forming sequences at gene promoters, acting as epigenetic-like structural modifications that modulate transcription and APE1-mediated G4 stabilization [53–55]. Up to now, whether analogous mechanisms may act at iM level, in particular at telomeres, is still unknown. The present study provides the first comprehensive characterization of APE1 ability to recognize telomeric iM structures and to process those containing AP-sites and proposes a mechanistic model in which APE1 and PCBP1 may cooperate to regulate iM dynamics, AP-site processing and telomere homeostasis.

We first established that the telomeric C-rich sequence used in this study (C-NAT) folds into a stable iM at acidic pH, as confirmed by CD spectroscopy and 1D NMR. Notably, the iMab antibody used in the SwitchSENSE approach was able to recognize and bind the iM at pH values up to 6.5, while the quencher approach uncovered that the stability is not conserved at pHs >6.0. This observation suggests that the iMab antibody exerts a stabilizing effect on the iM fold, extending its accessible pH range. The higher binding measured here, compared to the previously published values [10], likely reflect a higher sensitivity of the SwitchSENSE technology used for our assays.

Next, we observed that APE1 stably binds the native telomeric iM structure, with dissociation constants in the low nanomolar range for both wild-type protein and the N-terminal truncated mutant N∆33 (WT: 6.48 nM versus N∆33: 5.46 nM). This contrasts with the behavior observed for G4 structures, where the disordered N-terminal region of APE1 contributes substantially to binding [28, 29]. 2D NMR analysis revealed that the most important residues engaged in iM binding are clustered within and around α-helices 7 and 9 in the canonical DNA binding and catalytic domain. While 2D NMR analysis identified the APE1 residues most engaged in iM binding, the complementary contact surfaces on the iM structure could not be fully mapped, besides the fact that this goes beyond the scope of the present study. Indeed, complete structural characterization of the APE1-iM interface will require dedicated approaches, such as 3D NMR or X-ray crystallography and represent an important direction for future investigations. Real-time single-molecule visualization using the SMADNE approach with an endogenously-mEGFP-tagged APE1 cell-line (A549 cl.31) directly detected APE1 binding to iM structures in a near-physiological context, in the presence of potential competitors for both APE1 interaction and substrate binding. Moreover, as the A549 cl.31 cell line expresses APE1 at endogenous levels, these observations are free form potential biases due to protein overexpression. Our results provide the first evidence to our knowledge that iM setup is compatible with C-Trap, as well as support the feasibility of SMADNE analysis using endogenously labeled proteins. Together, these findings visualize APE1 engaging with iMs for the first time.

Moreover, by introducing a single AP-site at different positions within the iM, we found that iM folding is not abolished, consistently with previous reports [32], but produced position-dependent effects on structural stability. AP-sites embedded in the cytosine core (AP16, AP17) significantly reduced melting temperatures (AP16: 33°C, AP17: 28°C) compared to loop-substituted varients (AP14, AP20: 44°C) and destabilized the iM at lower pH values (AP16: 5.8, AP17: 5.7). This differential stability has mechanicistic consequences for the repair: the less stable core-substituted iM (AP16, AP17) were more susceptible to APE1 cleavage compared to AP14 and AP20. This strong cleavage difference could either indicate an easier unwinding to ss substrates, favoring the exposure of the AP-site to the processing activity by APE1, or a more similar configuration to a duplex, being part of the cytosine core, and this could better resemble APE1’s canonical target. Although the overall repair efficiency of APE1 toward AP-sites in the iM structure was substantially lower than on duplex DNA (1300-fold) and than on unstructured ssDNA substrates (3–7-fold), the position-dependent activity pattern reveals that APE1 repair ability is not uniform across the different damaged iM and is regulated by the structural context of the lesion.

A marked distinction between binding and catalytic activity emerged from the analysis of APE1 truncated mutant: while APE1^N∆33^ binds damaged iM with affinity comparable or even slightly higher to the wild-type protein, it is completely unable to cleave any of the damaged iM substrates, despite retaining proficient endonuclease activity on unstructured ss ODNs (lower than the APE1^WT^) and duplex substrates (comparable to the APE1^WT^). The higher binding affinity of APE1^N∆33^ toward damaged iMs might reflect the inability of this mutant to promote iM unwinding: without the N-terminal region, the protein stably engages the iM but cannot remodel the structure to expose the AP-site for catalysis, resulting in a prolonged and non-productive interaction. Whether the N-terminal region plays a role in facilitating the conformational rearrangement of the iM, required for endonuclease activity, remains an open question that future structural and single-molecule studies will need to address. Together, these results raise the possibility of a repair-indipendent role of APE1 at iM level, potentially contributing to stability regulation of these structures, analogous to the proposed epitranscriptional regulatory role of APE1 at oxidized G4 to control gene expression.

APE1 has been shown to interact with several hnRNP proteins [42] known to influence iM folding (e.g. hnRNP K, hnRNP A1, PCBP1 [9, 24, 25]). Among these, PCBP1, a member of the PCBP family implicated in both iM stabilization and genome integrity [11, 12, 20–22], was selected for further functional characterization, given its previously documented roles in the competing formation of iM and G4 structures [23]. We confirmed APE1/PCBP1 protein-protein interaction in HeLa and U2OS cellular lines by PLA. By implementing previous telo-ChIP data about APE1 [28, 47] with PCBP1-specific telo-ChIP and combining PLA with telomeric FISH probes, we confirmed that both proteins are localized at telomeres in U2OS cells and that their interaction also happens at these genomic loci. Furthermore, we observed that PCBP1 inhibits APE1 endonuclease activity on telomeric iM sequences *in vitro*. Competition assays indicate that PCBP1 strongly competes with APE1 for the substrate. This inhibitory relationship defines a regulatory axis in which the repair of AP-sites within iMs can be suppressed by iM-binding proteins, reinforcing the hyphotesis that AP-sites in iM might hold non-canonical regulatory roles.

At the cellular level, depletion of APE1 and PCBP1 produced opposite effects on telomere length in ALT-U2OS model: APE1 KO was associated with slightly longer telomeres, which could reflect a compensatory increase of homologous recombination activation in absence of efficient BER at telomeres. Instead, PCBP1 depletion reduced telomere length in both wild-type and APE1 KO models, suggesting an independent role for PCBP1 in supporting ALT-mediated elongation. Notably, previous attempts to detect telomere length changes in U2OS cells by TRF assay upon transient APE1 silencing were unsuccessful due to extensive length of ALT telomeres; here, we overcame this limitation by sonicating genomic DNA prior to restriction digestion, enabling the detection of small but significant changes. To validate these findings and assess if they can be generalized beyond the ALT context, we performed TRF analysis in HeLa cells, which carry shorter telomeres and rely on telomerase for their maintenance. Using a doxycycline-inducible APE1 KD system, we observed consistent telomere shortening upon APE1 depeletion across all conditions, whereas PCBP1 silencing alone did not significantly affect telomere length in this model. In a transient co-silencing approach, APE1 depletion reduced mean telomere length and PCBP1 silencing produced a comparabele modest reduction; importantly, co-depletion of both proteins did not result in an additive effect. The absence of PCBP1 effect on telomere length in telomerase-positive HeLa cells likely reflects telomerase-mediated compensation, which is unavailable in the ALT context of U2OS cells. Taken together, the opposite effects of APE1 and PCBP1 in U2OS cells and their convergent behavior in HeLa cells support a model in which the two proteins act coordinately to maintain telomere length homeostasis, with their relative contributions shaped by the telomere maintenance mechanism employed by each cell line.

An important molecular explanation for the functional role of this cooperation might reside in APE1/PCBP1 interaction with shelterin protein TRF2. We found that APE1 and PCBP1 interact with TRF2 in a mutually dependent manner: depletion of either protein abolishes the interaction of the other with TRF2, without affecting total TRF2 protein levels. This co-dependency is consistent with the formation of a trimeric APE1-PCBP1-TRF2 complex at telomeres, or alternatively with TRF2 relocalization upon the depletion of these proteins. Given that TRF2 is essential for telomere capping and suppression of ATM-mediated DNA damage signaling [48], and that APE1 depletion has been shown to reduce TRF2 telomeric localization [47], the interdependence of APE1 and PCBP1 for TRF2 interaction might represent a key node in the coordination of BER activity and sheletrin function at telomere ends. Further structural and biochemical studies are needed to deeply characterize this aspect.

To explore APE1-iM interplay dynamics in a cellular context, we examined the impact of APE1 and PCBP1 depletion on iM formation in U2OS cells using iMab immunofluorescence. APE1 KO was associated with a reduction of iMab foci, providing evidence about APE1 involvement in the regulation of iM formation *in vivo*. Strikingly, PCBP1 depletion produced the opposite effect, with a significant increase in iMab foci, consistent with the recently demonstrated role of PCBP1 as a selective iM-unfolding protein [49]. Together with the biochemical and biophysical data, these observations suggest that the APE1 and PCBP1 regulation of iM forming sequences might constitute a regulatory axis governing iM structural dynamics in a cellular context.

Both APE1 and PCBP1 depletion activated DDR in U2OS cells, but through distinct mechanisms. APE1 loss induced p21 upregulation without a concomitant increase in ${\mathrm{\gamma }}$H2AX phosphorylation, suggesting that APE1 influences p21 expression through p53 activation, independently of evident DSB accumulation, potentially reflecting replication stress or increase of BER intermediates. PCBP1 depletion, in contrast, induced both ${\mathrm{\gamma }}$H2AX and p21 upregulation, consistent with a more upstream effect on the ATM-p53-p21 axis in this specific model. Strikingly, simultaneous depletion of both proteins reduced phosporilation of ${\mathrm{\gamma }}$H2AX and p21 levels, which could indicate a possible radical alteration of DNA damage signaling when they are both absent. These observations indicate that APE1 and PCBP1 operate at distinct nodes of the DDR and their combined activity is required for canonical damage response at telomeres.

Together, our findings support a mechanistic model in which APE1 and PCBP1 may jointly regulate the structural dynamic and repair of telomeric iM structures. In this model: (i) APE1 binds iM independently of its N-terminal region; (ii) the N-terminal region is required for AP-site processing within the iM, likely driving the structural unwinding necessary for cleavage; (iii) PCBP1 competes with APE1 for iM binding, inhibiting the repair of damaged iM and leading to potential accumulation of BER intermediates with potential regulatory roles; (iv) APE1, PCBP1, and TRF2 form an interdependent complex at telomeres, coordinating iM dynamics with shelterin functions and telomere length control. Our study not only provides a detailed characterization of an underexplored aspect of APE1, beyond the BER pathway and canonical DNA repair but, due to the widespread distribution of iM-forming sequences in the human genome and their enrichment at gene regulatory regions, also opens new avenues for translational applications in biology and medicine that go beyond telomere biology.

## Supplementary Material

gkag686_Supplemental_File

## Data Availability

All study data are included in the article and/or supporting information. The raw data underlying this article are available in Open Science Framework (OSF) at https://doi.org/10.17605/OSF.IO/DV24S. Due to extensive data size, the raw single-molecule imaging data are available via an approval process directly from the corresponding author.
