# Supplementary material for: APE1 binds and processes abasic sites present in i-motif DNA and cooperates with PCBP1 in maintenance of telomeric stability
Source: Nucleic Acids Res. 2026 Jul 8;54(13):gkag686. doi: 10.1093/nar/gkag686 (PMC13343191; doi:10.1093/nar/gkag686)
Supplement: gkag686_Supplemental_File [file gkag686_supplemental_file.docx]

**Supplementary Information**

**MANUSCRIPT TITLE**

**APE1 binds and processes abasic sites present in i-motif DNA and cooperates with PCBP1 in maintenance of telomeric stability**

**AUTHORS**

Alessia Bellina^1^, Matilde Clarissa Malfatti^1,2,*^, Tobias Obermann^3^, Kayla Mae Grooms^3^, Andreas Gjøsæther^3^, Zahraa Othman^4^, Gilmar Salgado^4^, Daniela Marasco^5^, Antonella Virgilio^5^, Veronica Esposito^5^, Giulia Antoniali^1^, Catia Mio^8^, Matteo Pivetta^8^, Magnar Bjørås^3,6,7^, Barbara van Loon^3^, Gianluca Tell^1,*^

^1^ Laboratory of Molecular Biology and DNA repair, Department of Medicine (DMED), University of Udine, Piazzale Massimiliano Kolbe 4, 33100, Udine, Italy.

^2^ Fondazione Italiana Fegato - ONLUS, Liver Cancer Unit, Trieste, Basovizza 34149, Italy.

^3^ Department of Clinical and Molecular Medicine, Faculty of Medicine and Health Sciences, Norwegian University of Science and Technology, 7491 Trondheim, Norway

^4^ Department of Life Sciences and Technology for Health, ARNA laboratory, INSERM U1212, CNRS, UMR 5320, University of Bordeaux, Bordeaux F-33076, France.

^5^ Department of Pharmacy, University of Naples Federico II, Via D. Montesano 49, 80131, Naples, Italy.

^6^ Centre for Embryology and Healthy Development, University of Oslo, Oslo, 0373, Norway

^7^ Department of Microbiology, Oslo University Hospital and University of Oslo, Oslo, 0424, Norway

^8^ Department of Medicine (DMED), University of Udine, Piazzale Massimiliano Kolbe 4, 33100, Udine, Italy

^*^ To whom correspondence should be addressed. Email: [gianluca.tell@uniud.it](mailto:gianluca.tell@uniud.it)

Correpondence may also be addressed to Matilde Clarissa Malfatti. Email: [matilde.malfatti@uniud.it](mailto:matilde.malfatti@uniud.it); matilde.mafatti@fegato.it

**Supplementary Methods**

**RNA extraction and quantitative Reverse Transcriptase‑PCR (qRT‑PCR)**

RNA isolation was performed on 1.5×10^6^ A549 wild-type cells and A549 APE1-GFP cells using the “NucleoSpin^®^ RNA” kit (Machery-Nagel; 740955.250) according to the manufacturer’s instructions.

One microgram of total RNA was reverse transcribed using the SensiFAST cDNA synthesis kit (Bioline, London, UK), according to the manufacturer’s instructions. The following sequences of primers were used: *APEX1* For: 5’- CCTGGACTCTCTCATCAATACTGG-3’, *APEX1* Rev: 5’- AGTCAAATTCAGCCACAATCACC-3’, *GAPDH* For: 5’- CCCTTCATTGACCTCAACTACATG-3’, *GAPDH* Rev: 5’- TGGGATTTCCATTGATGACAAGC-3’. qRT-PCR was performed with a CFX96 Real-Time System (Bio-Rad) using SensiFAST SYBR No-ROX kit (Bioline, London, UK).

**Immunofluorescence and live-cell imaging**

For immunofluorescence 80000 cells were fixed in 4% paraformaldehyde, then washed with PBS 1× and permeabilized with 0.25% Triton X-100 in PBS 1× for 5 min. After washing with PBS 1× and blocking for 1 h with 10% FBS in Washing Buffer (10 mM Tris HCl pH7.4, 150 mM NaCl and 0.01% Tween 20), cells were incubated with APE1 primary antibodies (Novus; NB 100-101) diluted 1:100 in blocking solution for 3 h at 37 °C. After several washes in Washing Buffer 1×, cells were incubated with labelled secondary antibodies Alexa Fluor® 488 (goat anti-rabbit IgG 111-545-003 Jackson ImmunoResearch, West Grove, PA, USA) 1:400 in blocking solution for 2 h at room temperature. Cells were washed and mounted using *FluoroshieldTM with DAPI* (Sigma; 1002788770) and fixed with polish. Cells were visualized through a Leica TCS SP8 laser-scanning confocal microscope (Leica Microsystems, Wetzlar, Germany).

For Live-cell imaging, 1× 10^4^ A549 wild-type and selected clone cells were plated in an 8-well chamber (Nunc LabTek chamber slide, Merck) and grown at 37°C and 5% CO2 using *RPMI 1640* complemented medium. After 24 hours each well was washed with PBS 1× and stained using *Hoechst-RPMI 1640* complemented medium and left to incubate for one hour at 37°C. Afterwards, cells were washed and visualized through a 100X objective of a Leica TCS SP8 laser-scanning confocal microscope (Leica Microsystems, Wetzlar, Germany).

**Cell cycle analysis**

Total 1× 10^6^ cells were harvested and resuspended in 1 ml of cold 70% ethanol in PBS 1× at -20°C for 1 hour. Fixed cells were washed three times to remove ethanol and permeabilized with 100 µL PBS Triton X-100 0.1%, 10 µg/mL RNase A and 50 µg/mL Propidium Iodide in the dark for 30 minutes. Cells were then analyzed with “Attune NxT^®^ Flow Cytometer” machine. A minimum of 3× 10^4^ cells were measured for each experimental condition. Analyses of the results were performed using FlowJo.

**Cell viability and proliferation assay**

Cell viability was measured using the 3 (4 5 dimethylthiazol 2 yl) 5 (3-carboxymethoxyphenyl) 2 (4-sulfophenyl) 2*H*-tetrazolium salt (MTS) assay (Celltiter 96 Aqueous One solution cell proliferation assay, Promega) on cells grown in 96-well plates. In detail, 4000 cells were plated on 96-wells and were allowed to attach to the plate for 24 h. The day after, cells were treated separately with different amounts of *MMS* (Merck KGaA; 129925) for 8 hours, or *CDDP* (Merck KGaA, P4394), for 24 hours. After treatment, the MTS solution was added to each well, and the plates were incubated for 2 h at 37 °C. Absorbance was measured at 490 nm using a multiwell plate reader (Synergy H1, Agilent). All experiments were run at least in triplicate. The values were standardized to wells containing media alone and the cell viability was expressed as a fold change compared to the DMF-treated cells.

**
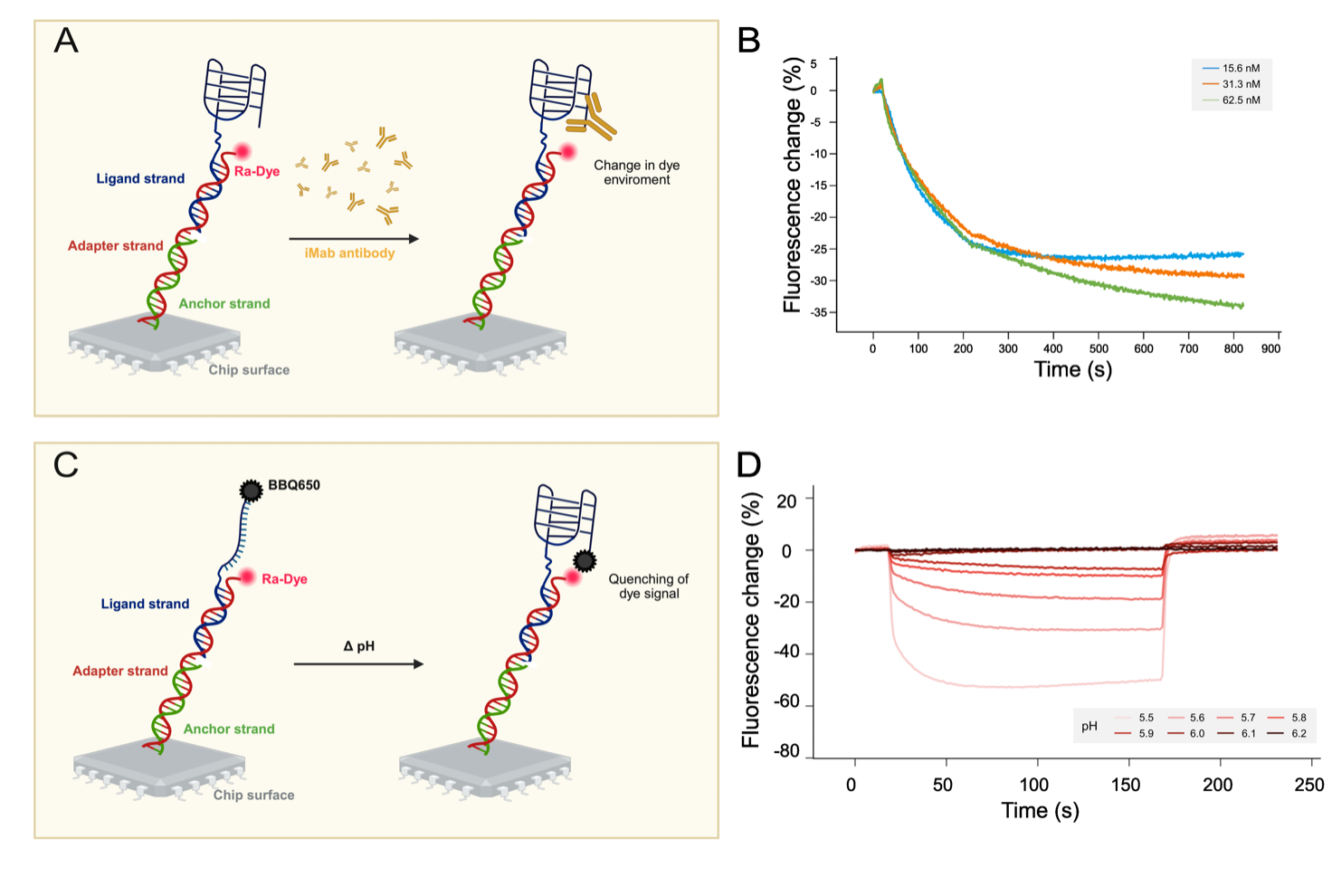
**

**Figure Supplementary 1.** A) Schematic representation of i-motif recognition by iMab antibody performed on SwitchSense. The anchor strand is indicated in green, the adapter strand in red, derivatized at its end with Ra-Dye, and the ligand strand in blue, with the iM structure at its end. The binding of the iMab antibody induces a change in the dye environment. B) Real-time fluorescence signals and fits of a representative experiment measuring the association and dissociation phases of iMab at different concentrations towards immobilized C-NAT at pH 5.5. Time (expressed in s) and Fluorescence change (expressed in %) are reported on the x- and y- axis, respectively. C) Schematic representation of i-motif formation assay through the quencher approach, performed on SwitchSense. The anchor strand is indicated in green, the adapter strand in red, derivatized at its end with Ra-Dye, and the ligand strand in blue, with the iM structure at its end, derivatized with BBQ650. The iM folding is monitored through the injection of buffers with increasing pH and by measuring the dye fluorescence. D) Real-time fluorescence signals of C-NAT derivatized with a quencher, after the injection of buffers with increasing pHs. Time (expressed in s) and Fluorescence change (expressed in %) are reported on the x- and y- axis, respectively. Curves referring to buffers with different pHs are represented in different colors.


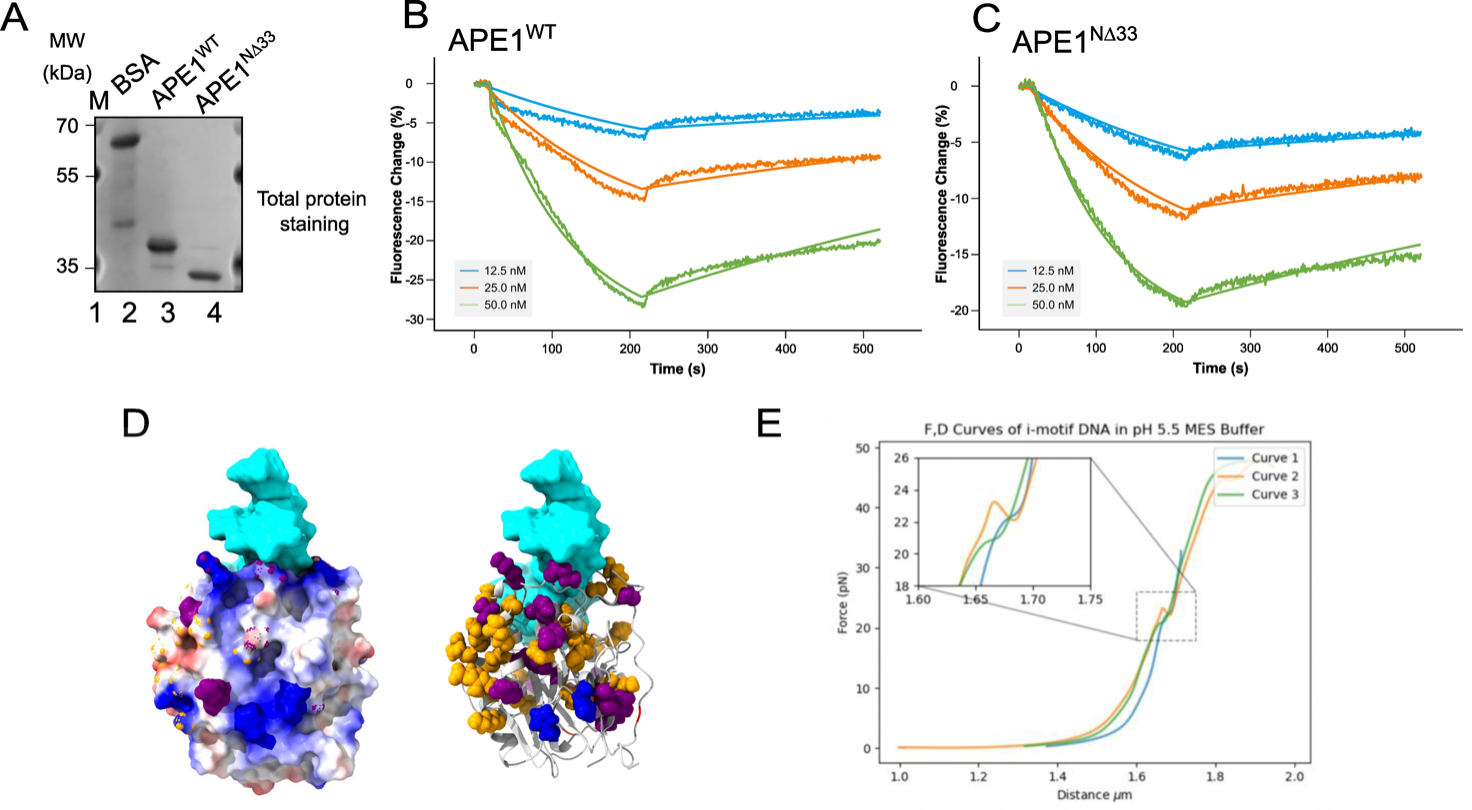


**Figure Supplementary 2.** A) Total protein staining showing the loading of equal amounts of the recombinant proteins BSA, APE1^WT^, APE1^N∆33^. On the sides, the electrophoretic marker (M) is loaded, and the different molecular weights (MW) are indicated and expressed in kDa. B-C) Real-time fluorescence signals and fits of a representative experiments measuring the association and dissociation phases of APE1^WT^ (B) and APE1^N∆33^ (C) at different concentrations towards immobilized C-NAT at pH 5.5. Time (expressed in s) and Fluorescence change (expressed in %) are reported on the x- and y- axis, respectively. D) Depiction of the side view of NMR-derived binding analysis on APE1 structure (PDB code: 1bix), relative to Figure 2D. E) Force-distance curves (n=3) of the 3x-C-NAT substrate. The unfolding events are zoomed in. Distance (expressed in µm) and Force (expressed in pN) are reported on the x- and y- axis, respectively.

**
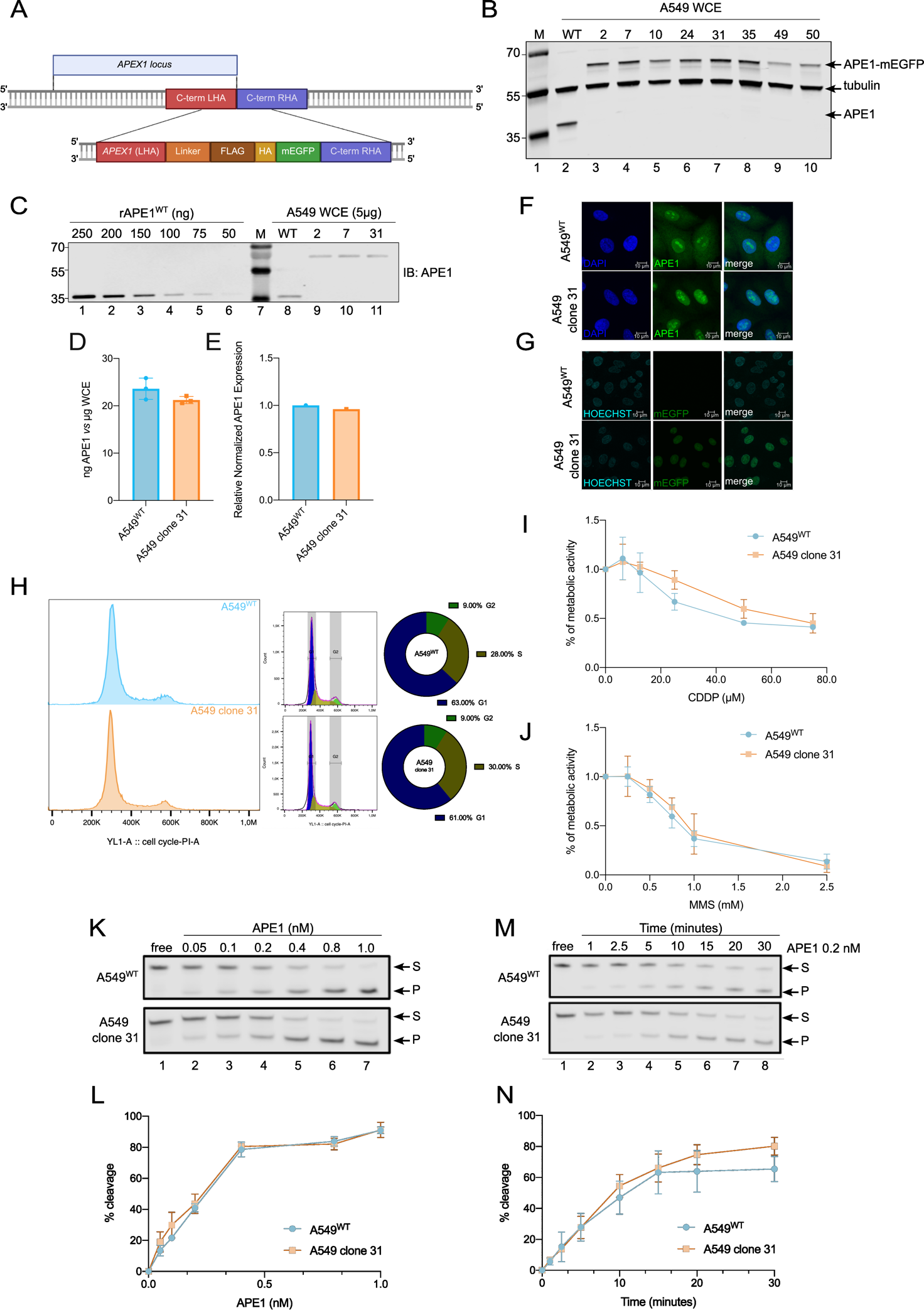
**

**Figure Supplementary 3.** A) Schematic representation of CRISPR–Cas9 technology used to generate the endogenous C-term tagging of APE1 in A549 cells. B) Representative western blot analysis comparing APE1-mEGFP levels to APE1^WT^. 20 µg of A549 WCE were loaded. Tubulin was used as a loading control. C-D) Quantification of APE1-mEGFP *via* rAPE1^WT^ standard curve. Protein quantification was estimated on a biological triplicate using 5 µg of A549 WCE and serial rAPE1^WT^ dilutions (250-50 ng). E) APE1-mEGFP gene expression quantification. *APEX1* expression levels were quantified *via* qPCR on a technical replicate and normalized to *GAPDH*. F-G) Immunofluorescence and live-cell imaging analysis for evaluating APE1-mEGFP localization. Both A549^WT^ and Clone 31 cells were fixed and stained with α-APE1 488 antibody and DAPI (F). For live-cell imaging, cells were stained with Hoechst (G). The μm scale is reported on the bottom left. H) Cell sorting analysis for Clone 31 cell-cycle phases quantification. G1, S and G2 phases distribution was measured on Clone 31 via Propidium Iodide (PI) using A549^WT^ as control. Percentage distributions are shown in the donut charts. I-J) Measurement of Clone 31 viability via MTS assay. Increasing concentrations of Cisplatin (CDDP) (I) and Methyl Methane Sulfonate (MMS) (J) were administered for both clone 31 and A549^WT^ to evaluate metabolic activity. DMF and RPMI 1640 were used as control vehicles, respectively. K-L) Representative AP-site incision assay and related estimation of APE1-mEGFP endonuclease activity. Increasing concentrations of APE1 (0.05-1.0 nM) from WCE of A549^WT^ and Clone 31 were tested to calculate endonuclease activities on a biological triplicate. The percentage of cleavage activity was estimated by comparing the Product (P) signal to the sum of both Substrate (S) and P signals. M-N) Representative AP-site incision assay and related estimation of APE1-mEGFP endonuclease kinetics. 0.2 nM APE1 from WCE of A549^WT^ and Clone 31 was incubated with substrate for increasing time points (1-30 minutes) to estimate endonuclease kinetics on a biological triplicate. The percentage of cleavage activity was estimated by comparing the Product (P) signal to the sum of both Substrate (S) and P signals.

**
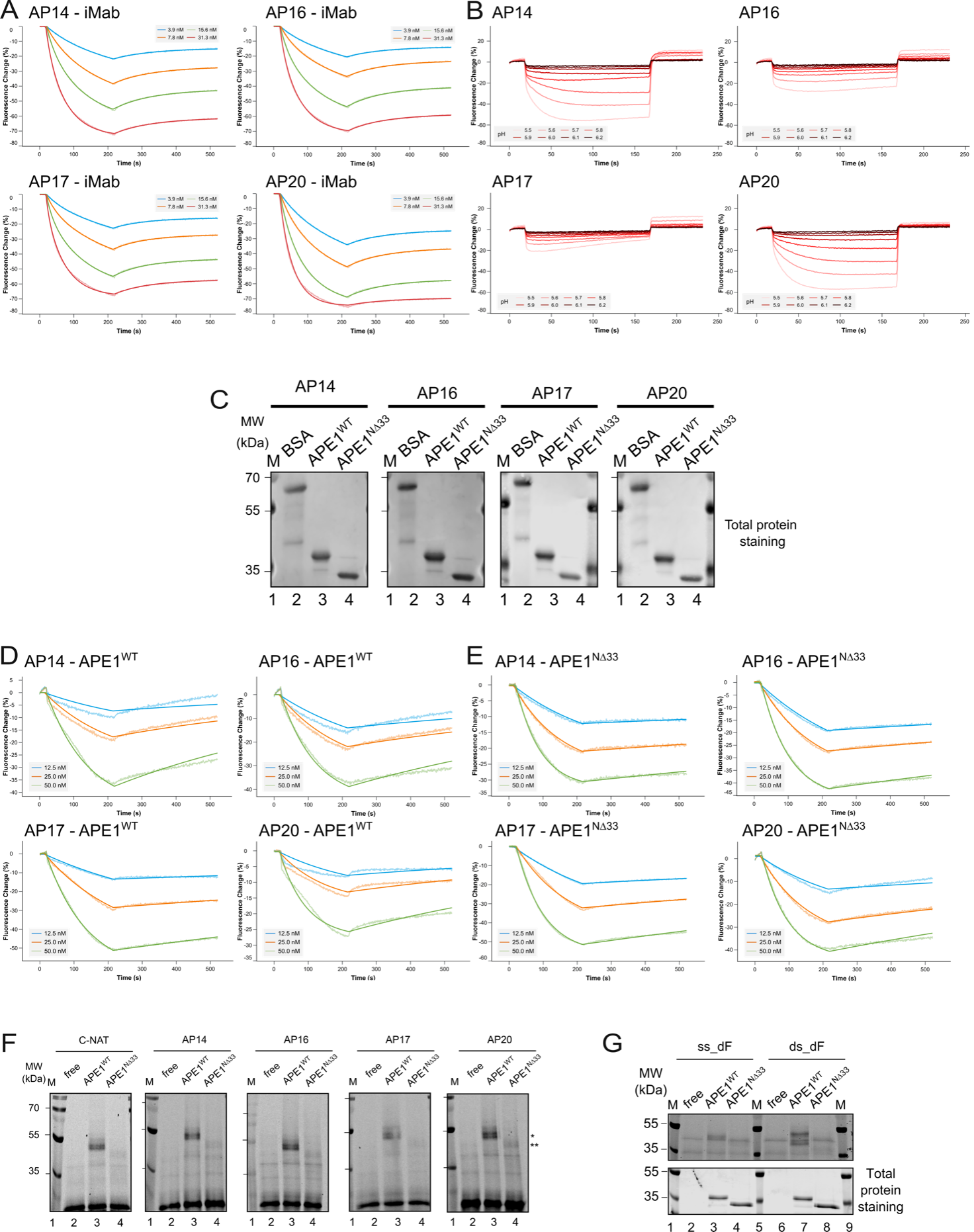
**

**Figure Supplementary 4.** A) Real-time fluorescence signals and fits of a representative experiment measuring the association and dissociation phases of iMab at different concentrations towards immobilized AP14, AP16, AP17 and AP20 at pH 6.5. Time (expressed in s) and Fluorescence change (expressed in %) are reported on the x- and y- axis, respectively. B) Real-time fluorescence signals of AP14, AP16, AP17 and AP20 derivatized with a quencher, after the injection of buffers with increasing pHs. Time (expressed in s) and Fluorescence change (expressed in %) are reported on the x- and y- axis, respectively. Curves referring to buffers with different pHs are represented in different colors. C) Total protein staining showing the loading of equal amounts of the recombinant proteins BSA, APE1^WT^, APE1^N∆33^. On the sides, the electrophoretic marker (M) is loaded, and the different molecular weights (MW) are indicated and expressed in kDa. D-E) Real-time fluorescence signals and fits of a representative experiment measuring the association and dissociation phases of APE1^WT^ (D) and APE1^N∆33^ (E) at different concentrations towards immobilized AP14, AP16, AP17 and AP20 at pH 5.5. Time (expressed in s) and Fluorescence change (expressed in %) are reported on the x- and y- axis, respectively. F) Representative crosslinking analysis with APE1^WT^ and APE1^N∆33^ recombinant proteins and the iM ODNs (25 nM). On the left, the electrophoretic marker is loaded, and the different molecular weights are expressed in kDa. G) Representative crosslinking analysis with APE1^WT^ and APE1^N∆33^ recombinant proteins and ss_dF and ds_dF ODNs (25 nM). On the left, the electrophoretic marker is loaded, and the different molecular weights are expressed in kDa. At the bottom, total protein staining of the gel, obtained with Coomassie, is reported.


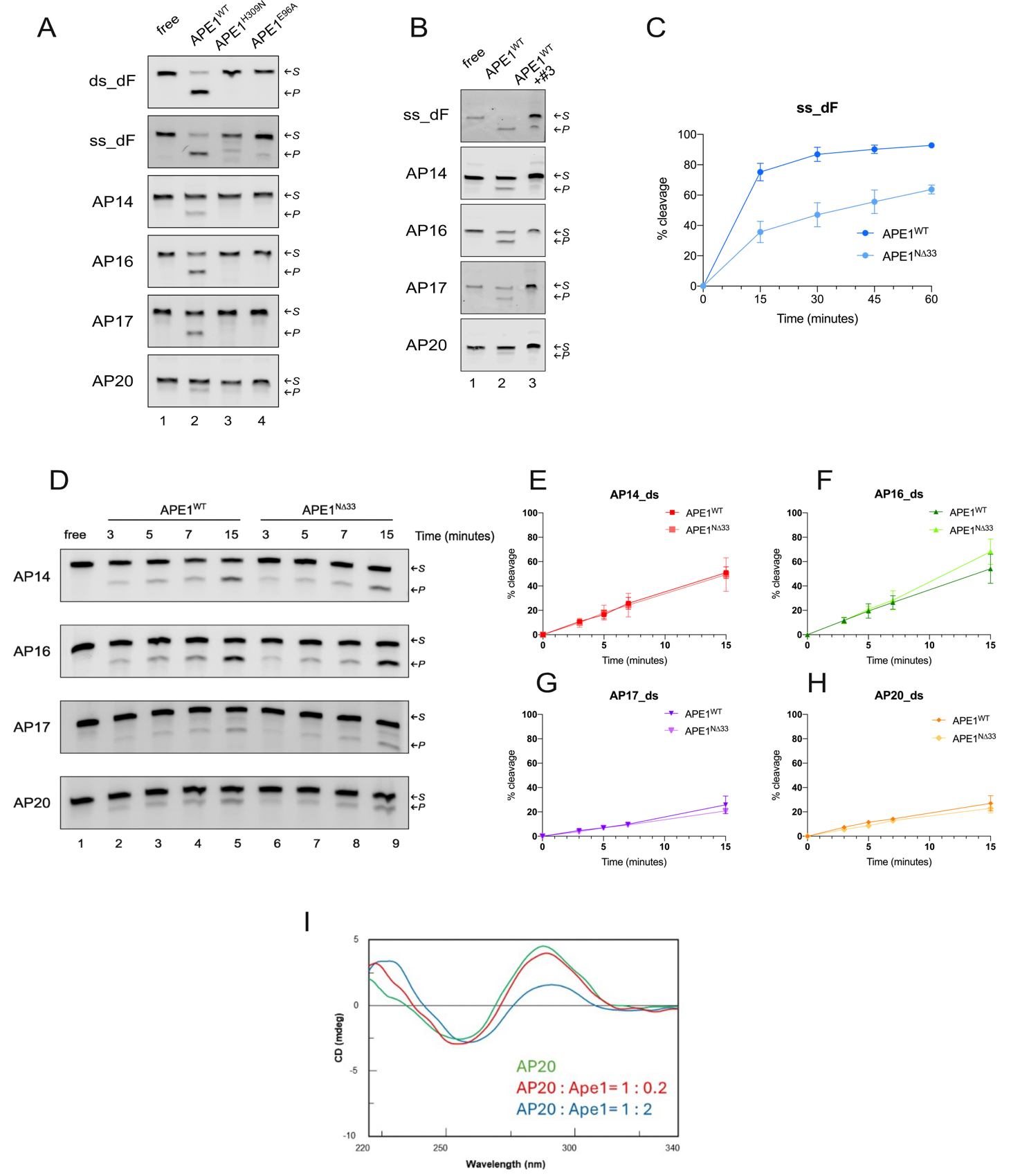


**Figure Supplementary 5.** A) Representative denaturing polyacrylamide gels of APE1^WT^ (lane 2), APE1^H309N^ (lane 3) and APE1^E96A^ (lane 4) cleavage activity performed on all substrates. “free” sample represents the control without protein (lane 1). On the right, the substrate and the product bands are indicated by two arrows. A constant dose of the wild-type protein and the indicated mutants (120 nM) were incubated with the respective oligonucleotide at 37°C for 1 hour, except for ds_dF, in which the substrate was incubated with 0.125 nM of each protein for 15 minutes. B) Representative denaturing polyacrylamide gels of APE1^WT^ cleavage activity performed on all substrates, in the absence (lane 2) or presence of compound 3 (#3) (lane 3). “free” sample represents the control without protein (lane 1). On the right, the substrate and the product bands are indicated by two arrows. A constant dose of the wild-type protein (120 nM) was incubated with the respective oligonucleotide at 37°C for 1 hour, either with or without #3 (5 mM). C) Relative graph illustrating the time-course kinetics activity of APE1^WT^ and APE1^N∆33^ recombinant proteins on ss_dF. Time (expressed in minutes) and percentage of cleavage (%) are reported on the x- and y- axis, respectively. Data are expressed as mean ± SD of three independent technical replicas. D) Representative denaturing polyacrylamide gels of cleavage analysis obtained on all ds_ substrates alone (lane 1, “free”), with APE1^WT^ protein (lanes 2-5) and with APE1^N∆33^ protein (lanes 6-9). On the right, the substrate and the product bands are indicated by two arrows. A constant dose of APE1^WT^ or APE1^N∆33^ (0.125 nM) was incubated with each oligonucleotide at 37°C, and the reactions were stopped at different time points, indicated upon the gel and expressed in minutes. E-H) Relative graph illustrating the time-course kinetics activity of APE1^WT^ and APE1^N∆33^ recombinant proteins on AP14_ds (E), AP16_ds (F), AP17_ds (G) and AP20_ds (H). Time (minutes) and percentage of cleavage (%) are reported on the x- and y- axis, respectively. Data are expressed as mean ± SD of three independent technical replicas. I) Overlay of CD spectra at 10°C in annealing buffer at 6 µM of AP20 at the indicated molar ratios of APE1^WT^.

**
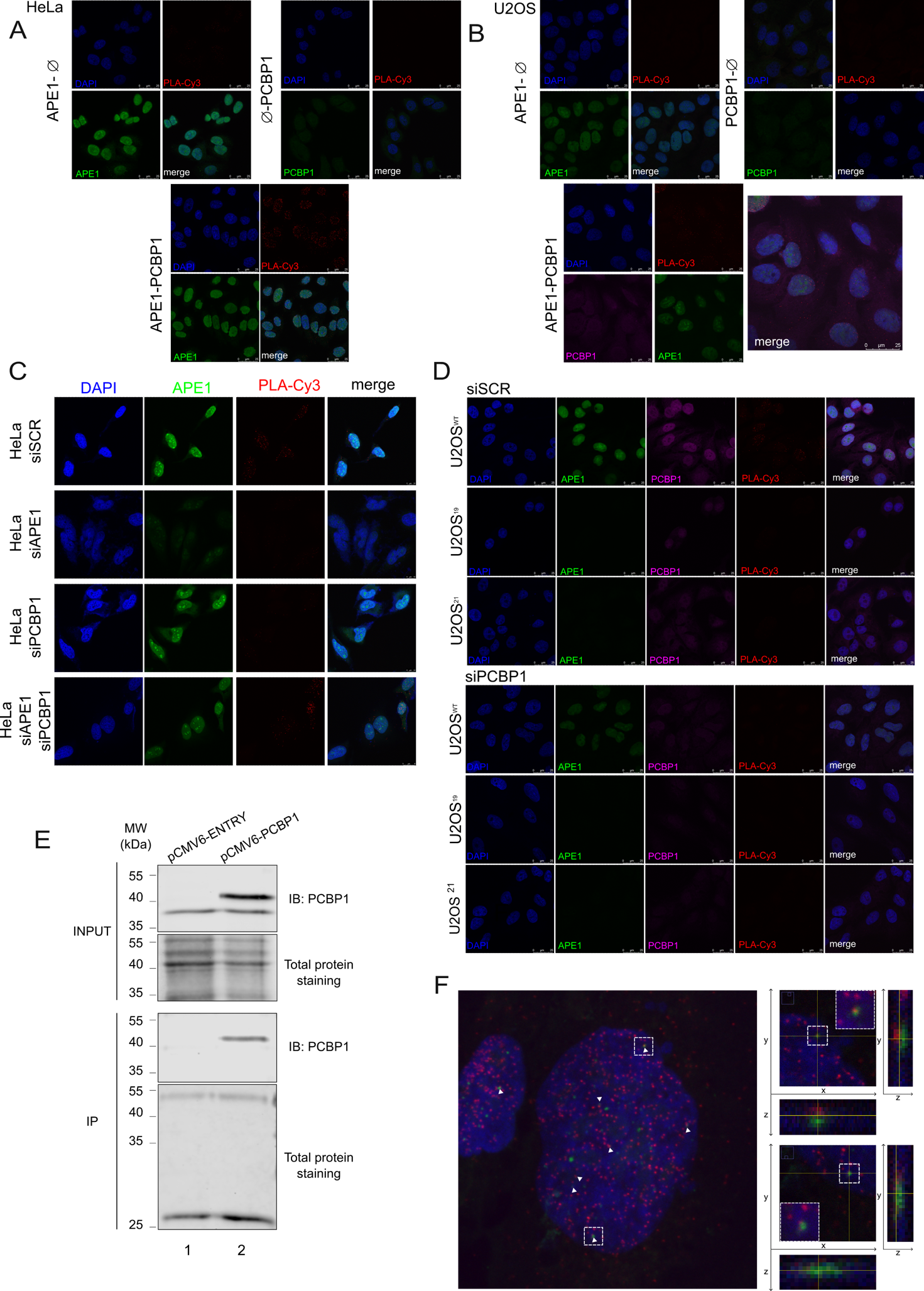
Figure Supplementary 6.** A) PLA analysis between APE1 and PCBP1 proteins in HeLa cells (APE1-PCBP1). Technical negative controls of PLA were obtained by omission of the antibody directed *versus* PCBP1 (APE1-Ø) or *versus* APE1 (Ø-PCBP1). APE1 staining was obtained with rabbit-488 (green) secondary antibody, while the nuclear staining, obtained with DAPI, is in blue. PLA dots are in red (Cy3-555). The merge panel shows the overlay between the four channels and reports the scale bar expressed in μm. B) PLA analysis between APE1 and PCBP1 proteins in U2OS cells. Technical negative controls of PLA were obtained by omission of the antibody *versus* PCBP1 (APE1-Ø) or *versus* APE1 (Ø-PCBP1). APE1 and PCBP1 staining were obtained with rabbit-488 (green) and mouse-633 (magenta) secondary antibodies, while the nuclear staining, obtained with DAPI, is in blue. PLA dots are in red (Cy3-555). The merge panel shows the overlay between the four channels and reports the scale bar expressed in μm. C) PLA reactions for HeLa cells transiently silenced for APE1 (siAPE1), PCBP1 (siPCBP1), or both proteins. Scramble control is also reported (siSCR). PLA spots (in red) show the interaction between APE1 and PCBP1 proteins. D) PLA reactions for U2OS clones, respectively U2OS^WT^, U2OS^19^ and U2OS^21^, transiently silenced for PCBP1. Scramble controls are also reported. PLA spots (in red) show the interaction between APE1 and PCBP1 proteins. E) Representative Western blot analysis relative to telo-ChiP experiment showing PCBP1 on total U2OS cell extracts (INPUT) and on the immunoprecipitated material (IP). The total protein staining, obtained by Revert700, is reported below each PCBP1-stained panel. The different molecular weights are indicated in kDa on the left side of the panels. F) Representative PLA analysis of the interaction between APE1 and PCBP1 proteins in U2OS cells (PLA dots in red), combined with telo-FISH (FISH dots in green). The merged panel shows the maximum projection of the overlay between the three channels, including the nuclear DAPI staining in blue. An arrow indicates the co-localized dots. On the right, the orthogonal views of the squared dots are reported.

**
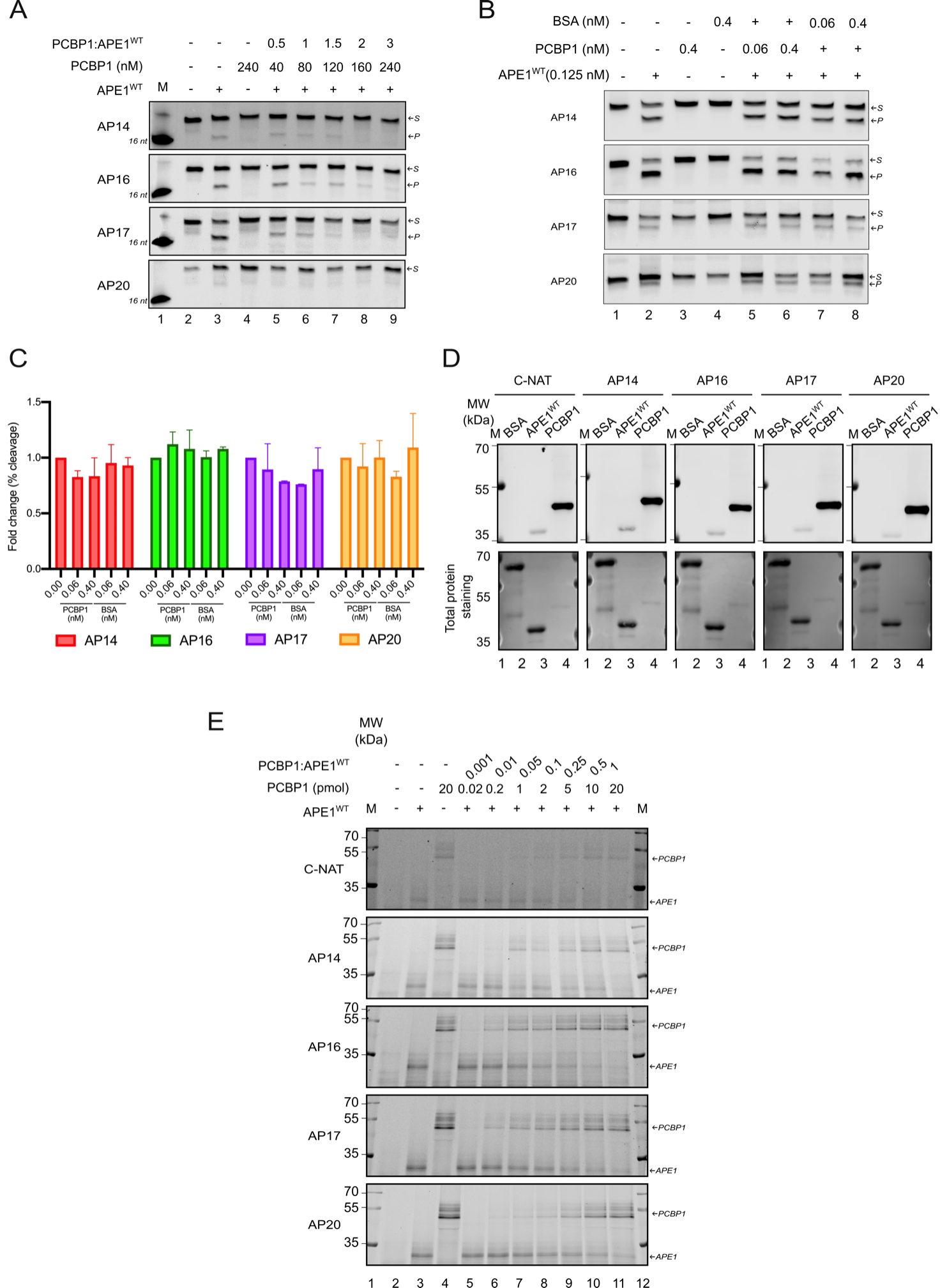
**

**Figure Supplementary 7.** A) Representative denaturing polyacrylamide gels of APE1^WT^ cleavage activity performed on all substrates (lane 3, 5-7), modulated by the co-incubation with PCBP1 (lane 5-7). Lane 2 is a control without any proteins. Lane 4 is a control with only the substrate and PCBP1. Lane 1 indicates a DNA fragment with a known length (16 nt). On the right, the substrate and the product bands are indicated by two arrows. The ODNs were pre-incubated with increasing amounts of PCBP1 (indicated upon the gel and expressed in nM) for 90 minutes at 4°C. A constant dose of APE1^WT^ (80 nM) was then added to the reactions and incubated at 37°C for 60 minutes. B) Representative denaturing polyacrylamide gels of cleavage analysis obtained on all substrates alone (lanes 1), with APE1^WT^ protein only (lanes 2), with PCBP1 and BSA only (lanes 3 and 4), with PCBP1 co-incubated with APE1 (lanes 5-6) and BSA co-incubated with APE1 (lanes 7-8). On the right, the substrate and the product bands are indicated by two arrows. The ODNs were pre-incubated with a variable dose of PCBP1 or BSA (upon the gel, nM) for 90 minutes at 4°C. Next, a constant dose of APE1 (0.125 nM) was added to the reactions and incubated at 37°C for 15 minutes, when the reaction was stopped. C) Relative graph illustrating the fold change of APE1^WT^ recombinant protein activity after pre-incubation with different amount of PCBP1 or BSA on AP14 (red), AP16 (green), AP17 (purple) and AP20 (orange). PCBP1 and BSA concentrations (nM) and fold change of the percentage of cleavage (%) are reported on the x- and y- axis, respectively. Data are expressed as mean ± SD of three independent technical replicas. D) Representative SWB shows the binding between the telomeric iM ODNs (reported upon the gel) and recombinant APE1^WT^ and PCBP1 proteins. BSA was used as a negative control. Each protein was loaded on the SDS-PAGE gel, blotted on the membrane and then incubated with the respective fluorescent probe (5 pmol). On the bottom, total protein staining reporting the loading of the recombinant proteins BSA, APE1^WT^, PCBP1. On the sides, the electrophoretic marker is loaded, and the different molecular weights are expressed in kDa. E) Representative UV-crosslinking competition analysis with APE1^WT^ and PCBP1 recombinant proteins and native and damaged iM. On both sides of the gel, the electrophoretic marker is loaded, and the different molecular weights, indicated on the left side, are expressed in kDa.

**
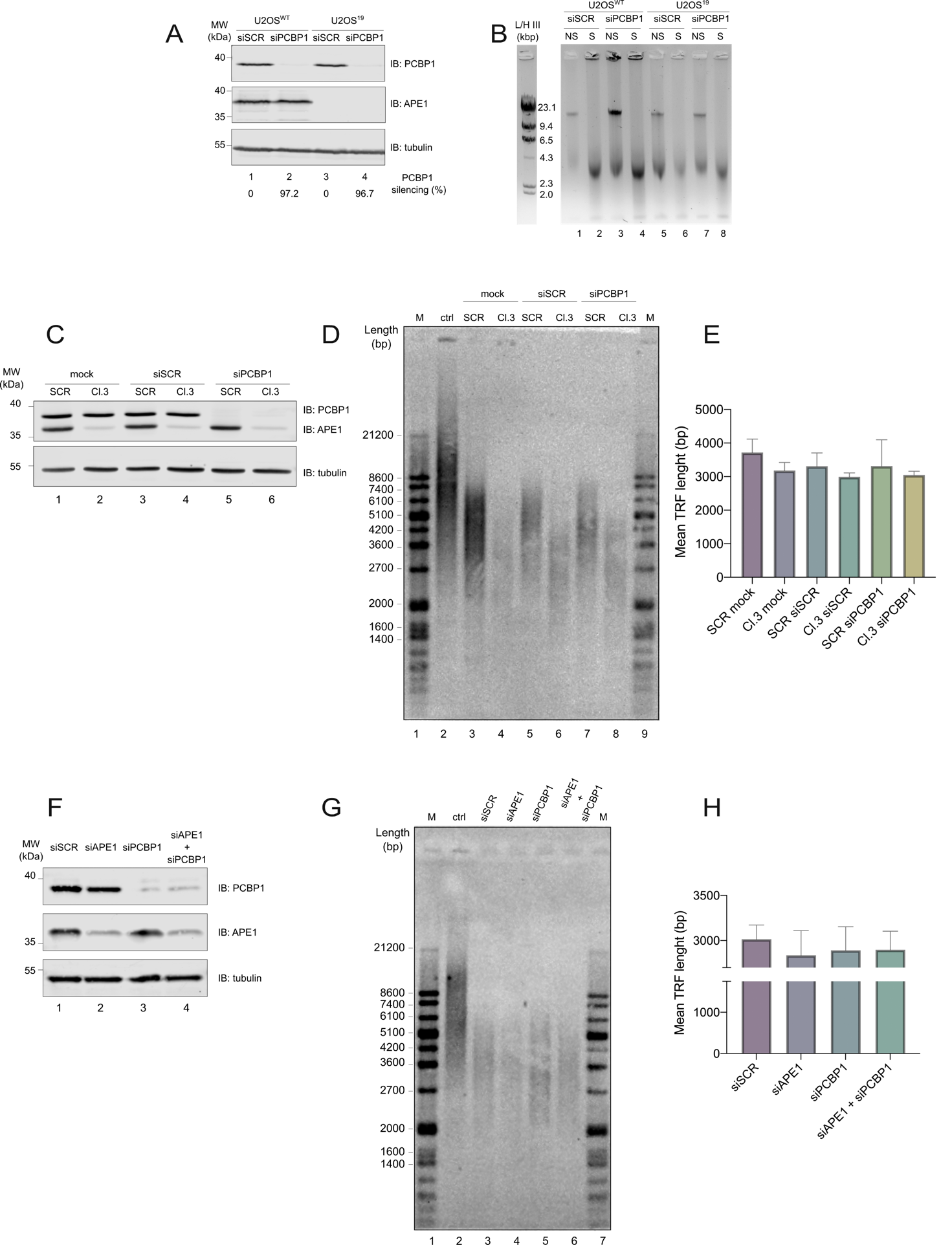
**

**Figure Supplementary 8.** A) Western blot analysis of PCBP1 and APE1 levels in U2OS^WT^, U2OS^19^ cell lines after PCBP1 transient silencing. Tubulin was used as normalizer. Molecular weight (MW, expressed in kDa) are reported on the left. The percentage of PCBP1 silencing is reported under each lane. B) Representative agarose gel of DNA extracted from U2OS^WT^, U2OS^19^ cell lines after PCBP1 transient silencing, non-sonicated (NS) or sonicated (S) for four cycles. On the left, the length marker Lambda/Hind III is reported and the length of each band is indicated in kbp. C) Western blot analysis of PCBP1 and APE1 levels in HeLa clone cells expressing (SCR) or knocked-down for APE1 (Cl.3) protein, either silenced or not for PCBP1 protein (siPCBP1 and siSCR, mock, respectively). Molecular weight (MW, expressed in kDa) are reported on the left. D) TRF assay was used to measure telomere length in HeLa cell clones expressing (SCR) or knocked-down for APE1 (Cl.3) protein, either silenced or not for PCBP1 protein (siPCBP1 and siSCR, mock, respectively), as indicated upon the gel. On the sides of the gel, the molecular weight marker, as provided by the kit, is loaded, and the length of each band is indicated and expressed as bp. E) Graph reporting TRF mean length of HeLa cell clones SCR and Cl.3 silenced for PCBP1 expression. Data are expressed as mean ± SD of two independent biological replicas. F) Western blot analysis of PCBP1 and APE1 levels in transiently silenced HeLa cells, respectively with siSCR, siAPE1, siPCBP1 and in combination, as indicated upon the gel. Molecular weight (MW, expressed in kDa) are reported on the left. G) TRF assay was used to measure telomere length in transiently silenced HeLa cells, respectively with siSCR, siAPE1, siPCBP1 and with a combination of siAPE1 and siPCBP1, as indicated upon the gel. On the sides of the gel, the molecular weight marker, as provided by the kit, is loaded, and the length of each band is indicated and expressed as bp. H) Graph reporting TRF mean length of HeLa cells silenced for APE1 and/or PCBP1 expression. Data are expressed as mean ± SD of two independent biological replicas.


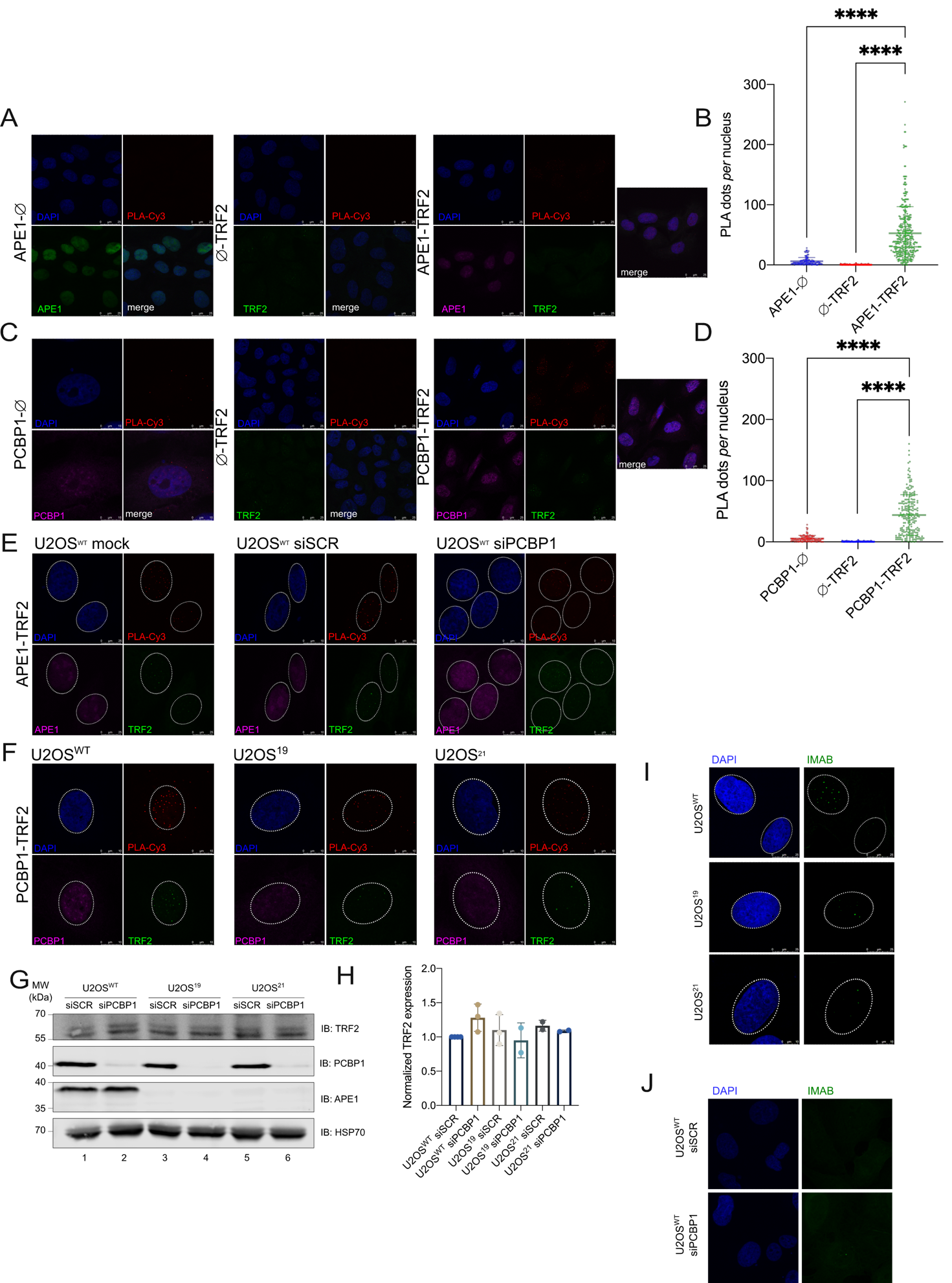


**Figure Supplementary 9.** A) PLA analysis between APE1 and TRF2 proteins in U2OS cells. Technical negative controls of PLA were obtained by omission of the antibody directed *versus* TRF2 (APE1-Ø) or *versus* APE1 (Ø-TRF2). TRF2 and APE1 staining were obtained with rabbit-488 (green) and mouse-633 (magenta) secondary antibodies, while the nuclear staining, obtained with DAPI, is in blue. PLA dots are in red (Cy3-555). The merge panel shows the overlay between the four channels and reports the scale bar expressed in μm. B) Graph depicting the number of TRF2-APE1 PLA dots *per* nucleus in U2OS^WT^, compared to technical negative controls. Average and standard deviation values are plotted (n = 1 for controls, n = 3 for APE1-TRF2). C) PLA analysis between PCBP1 and TRF2 proteins in U2OS cells. Technical negative controls of PLA were obtained by omission of the antibody directed *versus* TRF2 (PCBP1-Ø) or *versus* PCBP1 (Ø-TRF2). TRF2 and PCBP1 staining were obtained with rabbit-488 (green) and mouse-633 (magenta) secondary antibodies, while the nuclear staining, obtained with DAPI, is in blue. PLA dots are in red (Cy3-555). The merge panel shows the overlay between the four channels and reports the scale bar expressed in μm. D) Graph depicting the number of TRF2-PCBP1 PLA dots *per* nucleus in U2OS^WT^, compared to technical negative. Average and standard deviation values are plotted (n = 1 for controls, n = 3 for PCBP1-TRF2). Statistical analysis was performed using one-way ANOVA test. E) Single panels relative to the merge reported in Figure 6D. PLA dots are in red (Cy3-555), TRF2 and APE1 staining were obtained with mouse-488 (green) and rabbit-633 (magenta) secondary antibodies, while the nuclear staining, obtained with DAPI, is in blue. The scale is indicated and expressed in μm. F) Single panels relative to the merge reported in Figure 6F. PLA dots are in red (Cy3-555), TRF2 and PCBP1 staining were obtained with mouse-488 (green) and rabbit-633 (magenta) secondary antibodies, while the nuclear staining, obtained with DAPI, is in blue. The scale is indicated and expressed in μm. G) Western blot analysis of TRF2, PCBP1 and APE1 levels in U2OS^WT^, U2OS^19^ and U2OS^21^ cell lines after PCBP1 transient silencing. HSP70 was used as loading control and normalizer. Molecular weight (expressed in kDa) is reported on the left. H) Densitometric analysis of TRF2 expression levels, normalized to HSP70. Fold change values relative to U2OS^WT^ siSCR, arbitrary set to 1, are shown. Values are mean ± SD of two independent replicates. I) Representative immunofluorescence images of iMab antibody staining in U2OS^WT^, U2OS^19^ and U2OS^21^ cell lines. iMab foci are shown in green (rabbit 488 secondary antibody), while nuclei were stained with DAPI. The scale is indicated and expressed in μm. J) Representative immunofluorescence images of iMab antibody staining in U2OS^WT^, either silenced with a scramble siRNA (siSCR) or for PCBP1 protein (siPCBP1). iMab foci are shown in green (rabbit 488 secondary antibody), while nuclei were stained with DAPI.
